# Spatially Aware Pair Proposal for Panoptic Scene Graph Generation

**DOI:** 10.3390/s26134119

**Published:** 2026-06-30

**Authors:** Hanzhu Dai, Qiang Zhang, Binghao Wang, Mai Liu

**Affiliations:** 1 College of Automation, Jiangsu University of Science and Technology, Zhenjiang 212100, China; 232210503201@stu.just.edu.cn (H.D.); 232210305227@stu.just.edu.cn (B.W.); 2 Systems Science Laboratory, Jiangsu University of Science and Technology, Zhenjiang 212100, China; 3 College of Computer Science, Jiangsu University of Science and Technology, Zhenjiang 212100, China; 232241821506@stu.just.edu.cn

**Keywords:** Panoptic Scene Graph Generation, vision-sensor-based scene understanding, subject–object pair proposal, mask-level spatial modeling, grouped vector attention

## Abstract

Images captured by vision sensors provide visual evidence for scene understanding, including object appearances, pixel-level regions, and spatial relations among entities. Panoptic Scene Graph Generation (PSG) constructs structured scene representations by grounding visual entities with panoptic masks and predicting relationships among objects and regions. In pair-then-relation PSG pipelines, subject–object pair recall is critical to final triplet recall. However, existing pair proposal approaches mainly score candidate subject–object pairs based on object–query feature matching, while mask-derived spatial cues such as object locations, relative geometry, and local layouts remain underexplored. Consequently, ground-truth subject–object pairs may be excluded from the Top-$K_{r}$ proposals before relation decoding. To address this problem, this paper proposes a Spatially Aware Pair Proposal Model (SAPPM), which incorporates mask-derived soft centroids, relative geometry, and local-neighborhood context into pair scoring. SAPPM uses Grouped Vector Attention (GVA) to model local spatial interactions and introduces a spatially adaptive gating module to calibrate spatial-branch contributions. Experiments on the PSG dataset under the Scene Graph Detection (SGDet) protocol show that SAPPM achieves competitive performance, reaching 32.53 R@20 and 27.36 mR@20. These results indicate that SAPPM improves PSG performance by enhancing ground-truth pair coverage in the candidate proposal set.

## 1. Introduction

Images captured by vision sensors constitute a fundamental source of environmental perception for autonomous vehicles, mobile robots, and other intelligent sensor-driven systems. In these applications, perception modules are expected not only to detect objects or segment semantic regions, but also to support higher-level scene cognition for navigation, planning, and decision-making. Recent studies in sensor-based scene graph modeling have emphasized that comprehensive scene understanding requires the joint modeling of scene elements, spatial layouts, and inter-object relations, rather than isolated recognition of individual visual targets [1]. Scene Graph Generation (SGG) addresses this requirement by converting sensor-captured visual observations into structured semantic graphs, where entities are represented as nodes, and their semantic or spatial interactions are represented as directed edges, typically in the form of subject–predicate–object triplets [2]. Such relational scene representations provide an intermediate bridge between low-level visual perception and high-level environmental cognition, and have been applied to autonomous-driving scene understanding, embodied navigation, robotic action planning, traffic-scene retrieval, and intelligent surveillance [3,4,5,6,7]. However, conventional SGG mainly relies on bounding-box-level object representations, which limits its ability to describe irregular object extents, background regions, and fine-grained spatial structures in complex sensor-captured scenes. Panoptic Scene Graph Generation (PSG) further grounds visual entities with panoptic segmentation masks and models relations among foreground objects and background stuff regions, making it more suitable for mask-level structured scene understanding in robot and autonomous-driving environments.

With the development of panoptic segmentation, increasing attention has been paid to Panoptic Scene Graph Generation (PSG) [8]. Unlike conventional box-based SGG, PSG grounds visual entities with panoptic segmentation masks. It not only requires pixel-level modeling of foreground objects, i.e., things, but also incorporates background regions, i.e., stuff, and their relations with foreground objects into a unified scene-structure representation. Therefore, PSG advances scene graph generation from box-level visual relation prediction to mask-level structured scene understanding, providing a more complete representation of objects, regions, and their relations in complex scenes. For this task, Yang et al. constructed the PSG benchmark and proposed two Transformer-based baselines, PSGTR and PSGFormer. PSGTR directly predicts relation triplets, whereas PSGFormer separately models object queries and relation queries and composes final triplets through a prompting-like query matching mechanism [8]. Subsequently, Pair-Net shifted the research focus to candidate subject–object pair generation before predicate classification. It demonstrated that pair recall is closely associated with final triplet recall and proposed a pair-then-relation strategy that first generates candidate subject–object pairs and then performs predicate classification [9]. Subsequent PSG studies have improved relation prediction from complementary perspectives, including predicate semantic calibration and long-tailed relation modeling [10,11], mask-level relation representation and relation constraints [12,13], open-set relation prediction [14], fair ranking and evaluation [15], vision-language prompting [16], and graph- or semantics-enhanced relation modeling [17,18]. Overall, PSG research has evolved from task definition and end-to-end baseline construction to key issues such as candidate subject–object pair generation, long-tailed relation modeling, predicate semantic calibration, mask-level representation enhancement, open-set relation prediction, relation constraints, fair evaluation, and vision-language enhancement.

Although existing PSG methods improve relation prediction from different perspectives, the coverage of ground-truth subject–object pairs before relation decoding remains a critical factor affecting final triplet recall. Prior studies have modeled relations around subject–object pairs from the perspectives of relation-object matching, candidate pair generation, relation constraints, open-set relation estimation, and candidate ranking [8,9,13,14,15]. Among them, Pair-Net makes this issue explicit by estimating a pair proposal matrix and selecting Top-*K* subject–object pairs before relation fusion [9]. In this pair-then-relation pipeline, candidate subject–object pair ranking determines which entity pairs can enter the subsequent relation decoder. If a ground-truth subject–object pair is not included in the Top-$K_{r}$ candidate set, the relation decoder cannot further predict its predicate category. Therefore, the ranking quality of the pair proposal directly affects the final triplet recall.

**Figure 1:**
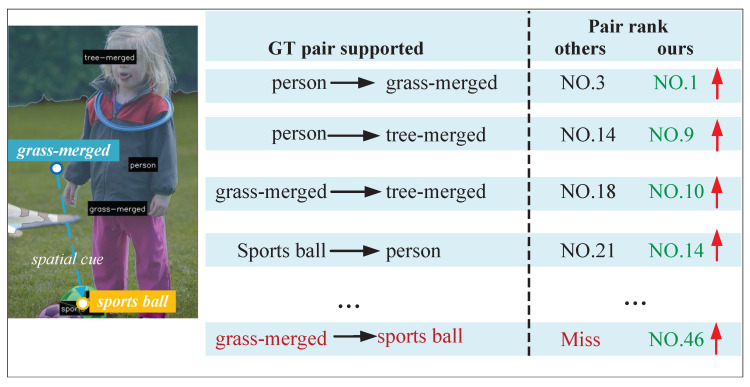
Motivation of spatially aware pair proposal. Some ground-truth subject–object pairs may receive low ranks or be missed before relation decoding when candidate ranking mainly relies on object-query matching. This motivates the use of mask-derived spatial cues to improve candidate pair ranking. Red text marks missed ground-truth pairs in the original candidate-pair ranking, green text marks the corresponding improved ranks after introducing spatial cues, and red upward arrows indicate rank improvements.

As shown in Figure 1, some ground-truth pairs may receive low ranks or be missed when candidate ranking mainly relies on object-query matching. For example, the ground-truth pair grass-merged → sports ball is missed in the compared result, whereas SAPPM ranks it as No. 46 and retains it in the Top-100 candidates. This phenomenon indicates that mask-derived spatial cues, such as soft centroids, relative geometry, and local layouts, can provide useful complementary information for candidate pair ranking. This motivates the introduction of mask-level spatial structure into pair proposal, so that candidate subject–object pair ranking can better correspond to the spatial configuration of the current image.

To address this candidate-pair retention bottleneck, this paper proposes a Spatially Aware Pair Proposal Model (SAPPM) for Panoptic Scene Graph Generation. Unlike pair proposal mechanisms that rank candidate pairs mainly according to object-level feature matching, SAPPM further exploits mask-derived object locations, relative geometric relations, and local layout information to introduce mask-level spatial structural cues into candidate subject–object pair scoring. By combining object representation matching with spatial structure modeling, SAPPM increases the likelihood that ground-truth subject–object pairs are covered by the selected Top-$K_{r}$ candidate set under the current scene layout, thereby providing a more reliable candidate basis for the subsequent relation decoder.

The main contributions of this paper are summarized as follows:

- We propose SAPPM, a spatially aware pair proposal framework that improves Top-$K_{r}$ ground-truth pair coverage by injecting mask-level spatial structure into pair ranking.
- We design a spatially aware pair-scoring module that estimates spatial anchors from predicted masks and incorporates mask-level spatial cues into pair proposal through relative position encoding, Grouped Vector Attention (GVA)-based local-neighborhood aggregation, and a distance-aware gate. This design enhances the spatial discriminability of the candidate subject–object pair ranking.
- We evaluate SAPPM on the PSG dataset under the Scene Graph Detection (SGDet) protocol. Experimental results show that SAPPM achieves competitive performance and outperforms the baseline under the same object parsing setting across R@K and mR@K metrics, indicating that mask-level spatial structure improves candidate pair coverage and benefits relation prediction.

## 2. Related Work

### 2.1. Scene Graph Generation and Panoptic Scene Graph Generation

Scene Graph Generation (SGG) aims to parse an image into a structured graph representation, where visual entities are treated as nodes and their semantic or spatial relationships are represented as edges. Early Visual Relationship Detection (VRD) and SGG methods typically followed a detect-then-classify paradigm: they first detected objects, enumerated directed subject–object pairs, and then predicted a predicate category for each pair to form subject–predicate–object triplets [2,19,20,21,22,23]. Subsequent studies improved relation prediction through context reasoning, graph-structured modeling, language priors, and category co-occurrence modeling, thereby advancing SGG from local candidate relation classification toward structured relation reasoning [2,24,25,26,27]. With the emergence of Transformer-based query modeling and end-to-end prediction paradigms, recent SGG methods have moved beyond the conventional detect-then-classify pipeline. These methods model visual relations through entity–relation joint representations, sparse triplet set prediction, object-query-based relation parsing, and graph-aware representation learning [28,29,30,31,32]. Despite these advances, relation prediction remains challenged by long-tailed predicate distributions, semantic ambiguity among predicate categories, and annotation bias. To mitigate these issues, existing studies have explored causal debiasing, resampling strategies, graph message passing, cognitive hierarchy modeling, noisy-label correction, class-balanced learning, and relation-attention reweighting [33,34,35,36,37,38]. Overall, SGG research has evolved from box-level candidate relation classification to end-to-end structured relation modeling. Despite this progress, most existing methods still rely primarily on bounding-box-level object representations, leaving fine-grained mask-level structures insufficiently modeled.

Panoptic Scene Graph Generation (PSG) extends scene graph generation from box-level object representation to mask-level scene understanding, requiring models to parse foreground objects, background stuff regions, and their relations based on panoptic segmentation masks [8]. Yang et al. established the PSG benchmark and introduced two Transformer-based baselines, PSGTR and PSGFormer, which respectively formulate PSG through direct triplet prediction and object–relation query matching [8]. Following this task definition, subsequent studies have improved PSG from several complementary directions. Pair-Net emphasized the upstream role of candidate subject–object pair generation and showed that pair recall strongly affects final triplet recall [9]. Other studies have addressed predicate-side difficulties such as semantic ambiguity, annotation bias, and long-tailed relation distributions through semantic prototype learning, adaptive supervision, and frequency-aware relation modeling [10,11]. Meanwhile, mask-level relation representation and relation constraints have been investigated through shape-aware features, curriculum learning, and constraint-aware relation modeling [12,13]. Further extensions have explored open-set relation prediction, fair ranking and evaluation, vision-language prompting, Transformer-based graph deduction, and semantic attention enhancement [14,15,16,17,18]. Overall, these studies have expanded PSG from benchmark construction and end-to-end baseline modeling to candidate-pair organization, predicate calibration, mask-level relation representation, open-set generalization, and evaluation-oriented refinement. Nevertheless, the effective coverage of ground-truth subject–object pairs before relation decoding remains a critical upstream factor that constrains final triplet recall.

### 2.2. Candidate Pairing and Relation Detection

In scene graph generation, the construction and filtering of candidate subject–object pairs define the search space for subsequent predicate classification. Since the number of object combinations grows rapidly with the number of detected entities, related studies have gradually shifted from exhaustive object-pair classification to more selective candidate relation modeling. Early methods mainly selected or modeled potential relation edges through relation proposal, graph pruning, and graph-structure-aware modeling, thereby reducing the interference of invalid object combinations in relation prediction [27,39,40]. Later studies further developed sparse and end-to-end paradigms for candidate relation organization. One line of work formulates relation detection as sparse triplet set prediction, where a limited number of relation queries directly predict subject–predicate–object triplets, avoiding dense enumeration over all object combinations [29,41]. Another line of work models interactions between object queries and relation queries, allowing relation candidates to be progressively formed during decoding and completing relation-object alignment through matching or attention mechanisms [28,31]. Other studies adopt fully convolutional relation fields or pixel-level association fields to integrate object localization and relation connectivity within a dense prediction framework, from which candidate relation structures can be recovered from response maps [30]. These methods move beyond the traditional pipeline of detecting entities, enumerating object pairs, and classifying predicates, indicating that candidate relation generation has become an important research problem in relation detection.

In PSG, Pair-Net further formulates this issue as candidate subject–object pair generation before relation decoding. Specifically, it uses a Pair Proposal Network (PPN) to generate and select candidate subject–object pairs, and then provides the resulting selected pairs as inputs to the relation decoder [9]. Later PSG studies have also examined candidate relation construction from the perspectives of mask matching, relation constraints, open-set relation existence estimation, and fair ranking [13,14,15]. Therefore, candidate subject–object pair generation forms a critical intermediate stage between pixel-level object parsing and relation decoding, motivating the use of mask-level spatial structure to improve pair proposal before predicate classification.

### 2.3. Spatial Geometric Modeling and Grouped Vector Attention

Spatial structure provides important cues for relation prediction in scene graph generation. Traditional box-level SGG methods commonly use subject/object bounding boxes, relative positions, union regions, or geometric encoding to describe the spatial layout between subject and object, thereby providing positional and shape-related information for predicate prediction [19,21,22,23,24,40]. With the development of attention mechanisms, spatial modeling has evolved from fixed geometric descriptors to learnable context aggregation, such as capturing spatial dependencies through global response relations, local window attention, sparse sampling, or reference points [42,43,44]. These studies suggest that both explicit geometric information and learnable spatial interactions can complement visual relation modeling. However, most of these methods are designed for bounding boxes, pixels, patches, or general visual features, and their spatial descriptions typically rely on rectangular boxes, union regions, or predefined sampling locations. This makes them difficult to directly adapt to panoptic-mask-based candidate subject–object pairing in PSG.

In irregular spatial data modeling, point cloud understanding methods further highlight the importance of local-neighborhood structure and relative position encoding. PointNet++ hierarchically aggregates point features through local neighborhoods in metric space. Point Transformer introduces self-attention and relative position encoding within local neighborhoods. Point Transformer V2 further proposes Grouped Vector Attention (GVA), which allows different channel groups to learn differentiated neighborhood weights and strengthens the modeling of local geometric interactions [45,46,47]. These studies indicate that neighborhood-based relative geometric aggregation provides an effective mechanism for modeling irregular spatial structures. Inspired by these neighborhood-based geometric aggregation mechanisms, this work investigates how mask-level spatial cues can be exploited at the pair proposal stage of PSG to enhance the spatial discriminability of candidate subject–object pair ranking.

## 3. Materials and Methods

### 3.1. Method Overview

In the pair-then-relation PSG framework, the quality of the candidate subject–object pair proposal plays a crucial role in final relation triplet prediction. The model first ranks all directed entity pairs and selects the Top-$K_{r}$ candidate subject–object pairs, and then performs predicate prediction based on these selected pairs. This process can be formulated as:

(1) $$P_{r}={\mathrm{TopK}}_{r}\left(U(S^{pair})\right),\hat{R}=\mathrm{Decoder}\left(P_{r},Q\right),$$

where $S^{pair}$ denotes the proposal matrix of candidate subject–object pairs, $P_{r}$ denotes the selected candidate pair set, and *Q* denotes the object query features. This formulation shows that the ranking quality of $S^{pair}$ directly affects which entity pairs participate in subsequent relation decoding. Therefore, constructing a more reliable pair proposal matrix before relation decoding provides an important basis for improving the coverage of ground-truth relation pairs in the Top-$K_{r}$ candidates and further improving final relation triplet prediction. To this end, we propose the Spatially Aware Pair Proposal Model (SAPPM), which introduces spatial structural information from panoptic masks into the candidate pair selection process. Panoptic masks not only correspond to pixel-level entity regions, but also contain mask responses that can be used to estimate entity positions. Based on this property, we first compute the soft centroid of each entity from mask predictions, and then construct relative geometry between entities accordingly. Subsequently, KNN is used to select local neighboring objects in the two-dimensional image plane, enabling spatial modeling to focus on local layout relations that are more relevant to the center entity.

**Figure 2:**
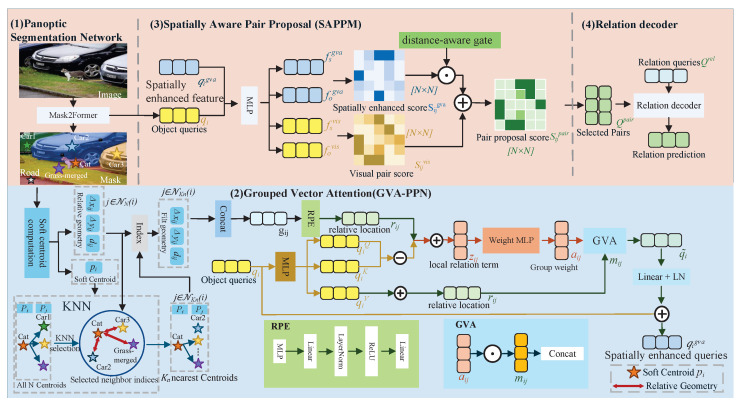
Framework of the Spatially Aware Pair Proposal Model (SAPPM).

As shown in Figure 2, the overall framework of SAPPM consists of a panoptic segmentation network, GVA-PPN, a spatially aware pair proposal module, and a relation decoder. First, the panoptic segmentation network outputs object queries and mask predictions and computes the soft centroid of each entity according to the mask response. Then, GVA-PPN constructs relative geometry and KNN neighborhoods based on the soft centroids and aggregates local spatial interaction information through relative position encoding and Grouped Vector Attention, obtaining spatially enhanced queries.

In the candidate pair selection stage, we construct a visual pair proposal matrix and a spatially enhanced pair proposal matrix using the original object queries and the spatially enhanced queries, respectively. The former preserves visual representations, category cues, and contextual information in object queries, while the latter supplements spatial structural information derived from soft centroids, relative geometry, and local layouts. Furthermore, a distance-aware gate adaptively fuses the two proposal matrices according to the spatial distance between the subject and the object, producing the final $S^{pair}$. Finally, the model selects the Top-$K_{r}$ candidate pairs based on $S^{pair}$, and the relation decoder further predicts predicate categories from these selected pairs to generate the final relation triplets.

### 3.2. Mask-Level Object Representation and Spatial Anchor Estimation

Let the object parsing branch output *N* object query features, denoted as

(2) $$Q={\left\{q_{i}\right\}}_{i=1}^{N},\;q_{i}\in R^{C},$$

where *N* denotes the number of object queries, and *C* denotes the channel dimension of each object query feature. In this work, N=100 and C=256. Each object query is associated with both a category prediction and a mask prediction. Unlike box-based relation modeling, PSG grounds visual entities with panoptic segmentation masks. Therefore, object spatial positions do not rely on explicit bounding box centers but can be directly estimated from pixel-level responses in the predicted masks. To enable the subsequent pair proposal process to exploit mask-level spatial cues, we extract a two-dimensional spatial anchor for each object from its mask logits.

Given the mask logits corresponding to the *i*-th object query,

(3) $$M_{i}\in R^{H\times W},$$

where *H* and *W* denote the spatial height and width of the mask prediction, respectively, and $M_{i}\left(u,v\right)$ denotes the raw mask response of the *i*-th object at pixel location (u,v). Since mask logits are not probability distributions, their values can be either positive or negative. If the raw logits are directly used as spatial weights for centroid computation, negative responses may cancel positive responses, thereby weakening the geometric meaning of the weighted average and potentially leading to unstable center estimation. Therefore, we first convert the mask logits into non-negative response weights:

(4) $${\tilde{M}}_{i}\left(u,v\right)=\max\left(M_{i}\left(u,v\right),0\right).$$

Here, ${\tilde{M}}_{i}$ denotes the non-negative mask response map of the *i*-th object. This operation preserves the positive response regions in the predicted mask and sets the spatial weights of non-positive response locations to zero, so that the subsequent centroid estimation is mainly determined by pixel locations that produce positive responses for the corresponding object query. It should be noted that ${\tilde{M}}_{i}$ is not a normalized mask probability, but an unnormalized response weight used for spatial weighted averaging.

We then compute the object soft centroid in the normalized image coordinate system. Let $x_{v}\in \left[0,1\right]$ and $y_{u}\in \left[0,1\right]$ denote the normalized horizontal and vertical coordinates corresponding to the *v*-th column and the *u*-th row, respectively. The two-dimensional spatial anchor of the *i*-th object is defined as

(5) $$c_{i}^{x}=\frac{\sum_{u,v}{\tilde{M}}_{i}\left(u,v\right)x_{v}}{\max\left(\sum_{u,v}{\tilde{M}}_{i}\left(u,v\right),\epsilon \right)},\;c_{i}^{y}=\frac{\sum_{u,v}{\tilde{M}}_{i}\left(u,v\right)y_{u}}{\max\left(\sum_{u,v}{\tilde{M}}_{i}\left(u,v\right),\epsilon \right)}.$$

Here, ϵ is a numerical stability term used to avoid a degenerate denominator in cases of low or empty responses. In the implementation, ϵ is fixed to $10^{-8}$, corresponding to the minimum value used when clamping the mask-response mass. Finally, the mask-level spatial anchor of the *i*-th object is represented as

(6) $$p_{i}=\left(c_{i}^{x},c_{i}^{y}\right)\in {\left[0,1\right]}^{2}.$$

For fragmented or multi-part masks, the centroid defined above corresponds to the first-order spatial moment of all positive mask responses. Let the positive response support of the *i*-th mask be decomposed into *L* disjoint components ${\left\{\Omega_{i}^{\left(l\right)}\right\}}_{l=1}^{L}$. For the *l*-th component, we define its response mass and local centroid as

(7) $$m_{i}^{\left(l\right)}=\sum_{\left(u,v\right)\in \Omega_{i}^{\left(l\right)}}{\tilde{M}}_{i}\left(u,v\right),\;p_{i}^{\left(l\right)}=\frac{\sum_{\left(u,v\right)\in \Omega_{i}^{\left(l\right)}}{\tilde{M}}_{i}\left(u,v\right)\left(x_{v},y_{u}\right)}{m_{i}^{\left(l\right)}}.$$

The resulting soft centroid can then be written as

(8) $$p_{i}=\frac{\sum_{l=1}^{L}m_{i}^{\left(l\right)}p_{i}^{\left(l\right)}}{\sum_{l=1}^{L}m_{i}^{\left(l\right)}}.$$

This formulation shows that, when an object is partially occluded or represented by several disconnected visible regions, the estimated anchor is determined by the relative response mass of these components. Components with larger spatial extent or stronger positive logits contribute more to the final anchor. Consequently, the anchor may fall between separated components when the visible support is highly fragmented. In SAPPM, this quantity is therefore interpreted as a compact mask-level spatial reference rather than a precise geometric center of the visible object region. It is used to construct local neighborhoods and relative geometric encodings for pair proposal, while the subsequent pair scoring is still performed over the complete N×N directed subject–object pair space. The original visual path and the distance-aware gate further moderate the contribution of this spatial reference during candidate-pair scoring.

The soft centroid does not serve as an additional localization supervision target. Instead, it serves as an intermediate representation that bridges object query features and the normalized image plane. Based on the spatial anchors ${\left\{p_{i}\right\}}_{i=1}^{N}$ of all objects, SAPPM constructs local neighborhoods in the normalized two-dimensional plane and further computes relative geometric relations between objects. In this way, the spatial structures implicit in mask predictions can be introduced into the pair proposal stage, providing mask-level geometric cues for candidate pair ranking and selection.

### 3.3. Local Neighborhood Construction Based on Relative Geometry

After obtaining the normalized mask-level soft centroids, SAPPM constructs object-level local neighborhoods based on ${\left\{p_{i}\right\}}_{i=1}^{N}$ to introduce local-neighborhood context into the subsequent GVA module. For any two object queries *i* and *j*, their soft centroids are denoted as $p_{i}=\left(c_{i}^{x},c_{i}^{y}\right)$ and $p_{j}=\left(c_{j}^{x},c_{j}^{y}\right)$, respectively. The relative displacement between them is defined as

(9) $$\Delta p_{ij}=\left(\Delta x_{ij},\Delta y_{ij}\right),$$

where

(10) $$\Delta x_{ij}=c_{i}^{x}-c_{j}^{x},\;\Delta y_{ij}=c_{i}^{y}-c_{j}^{y}.$$

The corresponding Euclidean distance is defined as

(11) $$d_{ij}=\sqrt{{\left(\Delta x_{ij}\right)}^{2}+{\left(\Delta y_{ij}\right)}^{2}}.$$

Based on the above soft centroids and pairwise distances, SAPPM constructs a local spatial neighborhood for each entity. As illustrated by the KNN selection part in Figure 2, all soft centroids are first represented as two-dimensional points on the normalized image plane. For an ordered candidate pair (i,j), we use the previously defined Euclidean distance $d_{ij}$ between their soft centroids for distance-aware gating.

(12) $$N_{K_{n}}\left(i\right)={\mathrm{KNN}}_{K_{n}}\left(p_{i},{\left\{p_{j}\right\}}_{j=1}^{N}\right)=\overset{\mathrm{smallest}}{\underset{j\in \{1,\ldots ,N\}}{\arg\mathrm{topK}}}d_{ij}.$$

Here, ${\mathrm{KNN}}_{K_{n}}\left(\cdot \right)$ denotes the index selection operation that returns the $K_{n}$ entity indices with the smallest $d_{ij}$. Taking the Cat example in Figure 2, the soft centroid of Cat serves as the center point, and the model searches all *N* centroids to find its nearest entities in the image plane. The selected neighboring entities, such as Car2 and Car3, form $N_{K_{n}}\left(\mathrm{Cat}\right)$. The model then computes relative geometry, including $\Delta x_{ij}$, $\Delta y_{ij}$, and $d_{ij}$, only between the center entity and the selected neighboring entities, and uses these geometric quantities as inputs to the subsequent RPE and GVA aggregation.

This KNN neighborhood enables GVA to aggregate spatial information around the local layout of the center entity. For example, neighboring entities such as Car2 and Car3 provide direct relative-position and proximity cues for Cat, allowing the spatially enhanced query of Cat to perceive the arrangement of nearby objects. Compared with treating all entities equally in spatial interaction, KNN concentrates the subsequent GVA aggregation on entities with closer spatial relations to the center entity, producing a more explicit local spatial representation. This representation is then used in the spatially enhanced pair proposal matrix and provides spatial structural evidence for candidate pairs involving the center entity and its nearby objects.

### 3.4. Relative Position Encoding

Raw coordinate differences are not well aligned with high-dimensional object features and therefore cannot be directly used for feature interaction. To address this mismatch, we feed the relative geometric vector between objects,

(13) $$g_{ij}=\left(\Delta x_{ij},\Delta y_{ij},d_{ij}\right),$$

into a learnable position encoding function to obtain the relative position embedding:

(14) $$r_{ij}=\mathrm{RPE}\left(g_{ij}\right),\;r_{ij}\in R^{C}.$$

RPE(·) consists of a linear projection, Layer Normalization, ReLU activation, and a subsequent linear projection. It maps low-dimensional relative geometric quantities to representations with the same dimensionality as the object features. Unlike directly using distances or coordinate differences as geometric scalars, $r_{ij}$ can participate in subsequent GVA computation as a feature vector, allowing relative displacement and distance information to be incorporated into subsequent neighborhood attention modeling.

### 3.5. Spatial Aggregation via Grouped Vector Attention

After obtaining the relative position embedding $r_{ij}$, the model updates object representations within the local neighborhood through Grouped Vector Attention (GVA). For the center object *i*, this process jointly uses its own feature $q_{i}$, the features of neighboring objects, and the corresponding relative position embeddings $r_{ij}$, thereby encoding local-neighborhood context into the object representation. For notational simplicity, we denote the local neighborhood of object *i* as N(i), where N(i) refers to the previously defined $K_{n}$-nearest-neighbor set $N_{K_{n}}\left(i\right)$. The model first maps object query features into query, key, and value representations:

(15) $$q_{i}^{Q}=\phi_{Q}\left(q_{i}\right),\;q_{i}^{K}=\phi_{K}\left(q_{i}\right),\;q_{i}^{V}=W_{V}q_{i}.$$

Here, $\phi_{Q}\left(\cdot \right)$ and $\phi_{K}\left(\cdot \right)$ consist of a linear projection, Layer Normalization, and ReLU activation, while the value branch uses a linear projection.

For a neighboring object j∈N(i), the model constructs a local relation term:

(16) $$z_{ij}=q_{j}^{K}-q_{i}^{Q}+r_{ij}.$$

This local relation term jointly encodes the feature-space difference and relative geometry between the center object and its neighboring object. Specifically, $q_{j}^{K}-q_{i}^{Q}$ characterizes their feature-space relation, while $r_{ij}$ provides relative displacement and distance information derived from object-center positions.

Unlike standard dot-product attention, GVA does not compress the relation between objects into a single scalar attention weight. Instead, it uses a grouped weight encoding function to generate group-wise neighborhood weights over *G* channel groups:

(17) $$\alpha_{ij}={\mathrm{Softmax}}_{j}\left(\psi (z_{ij})\right),\;\alpha_{ij}\in R^{G}.$$

Here, ψ(·) denotes the grouped weight encoding network, and the softmax operation is performed along the neighborhood dimension. Let the feature dimension *C* be divided into *G* channel groups, each with dimension C/G. Thus, $\alpha_{ij}$ does not assign a single neighborhood weight to the entire feature vector. Instead, each group-level weight is broadcast to its corresponding C/G-dimensional channel group, allowing neighboring objects to contribute differently across feature subspaces.

The model then adds the value feature of each neighboring object to the corresponding relative position embedding and aggregates them according to the group-wise neighborhood weights:

(18) $${\tilde{q}}_{i}=\sum_{j\in N(i)}\alpha_{ij}⊙\left(q_{j}^{V}+r_{ij}\right).$$

Here, ⊙ denotes group-wise multiplication over channel groups. In this aggregation, $q_{j}^{V}$ provides the appearance and contextual feature of the neighboring object, while $r_{ij}$ injects relative displacement and distance information. These two components jointly contribute to the local spatial context update of object *i*. Since different channel groups have independent neighborhood weights, the model can aggregate different types of neighborhood information in different feature subspaces, rather than relying on a single full-channel attention weight.

Finally, the aggregated neighborhood feature is passed through an output projection and added back to the original object query feature through a residual connection:

(19) $$q_{i}^{gva}=\mathrm{Proj}\left({\tilde{q}}_{i}\right)+q_{i}.$$

The resulting $q_{i}^{gva}$ preserves the semantic, appearance, and contextual information contained in the original object query feature through the residual connection, while incorporating spatial context encoded from the local neighborhood, relative positions, and object distances. The spatially enhanced object feature is then used for subject/object role projection in the GVA-enhanced spatial path and participates in candidate subject–object pair scoring.

### 3.6. Dual-Path Candidate Subject–Object Pair Scoring

After obtaining the spatially enhanced object feature $q_{i}^{gva}$, the model computes candidate subject–object pair scores based on both the original object feature $q_{i}$ and the spatially enhanced object feature $q_{i}^{gva}$. The dual-path scoring structure consists of a visual path and a GVA-enhanced spatial path. The visual path preserves semantic, appearance, and contextual co-occurrence cues encoded in the original object query features, while the GVA-enhanced spatial path introduces spatial context encoded from local neighborhoods and relative geometry.

The first path is the visual path, which computes role-aware feature matching scores from the original object query features. Specifically, the model applies subject role projection and object role projection to the object features:

(20) $$s_{i}^{vis}=\mathrm{Norm}\left(f_{s}^{vis}\left(q_{i}\right)\right),\;o_{j}^{vis}=\mathrm{Norm}\left(f_{o}^{vis}\left(q_{j}\right)\right),$$

where Norm(·) denotes L2 normalization. The visual score is then defined as

(21) $$S_{ij}^{vis}={\left(s_{i}^{vis}\right)}^{⊤}o_{j}^{vis}.$$

Since subject and object use different role projection functions, this path not only preserves the pairing cues provided by the original object features, but also explicitly distinguishes the functional roles of subject and object in directed subject–object pairs. Therefore, the resulting pair score matrix is directed, i.e., $S_{ij}^{vis}$ and $S_{ji}^{vis}$ are not equivalent.

The second path is the GVA-enhanced spatial path, which computes the GVA-enhanced spatial score from the spatially enhanced object features. This score can be viewed as a geometry-aware pair score because its input features encode local-neighborhood context and relative geometry:

(22) $$s_{i}^{gva}=\mathrm{Norm}\left(f_{s}^{gva}\left(q_{i}^{gva}\right)\right),\;o_{j}^{gva}=\mathrm{Norm}\left(f_{o}^{gva}\left(q_{j}^{gva}\right)\right),$$

(23) $$S_{ij}^{gva}={\left(s_{i}^{gva}\right)}^{⊤}o_{j}^{gva}.$$

Different from the visual path, which uses the original object features, the input $q_{i}^{gva}$ of the GVA-enhanced spatial path has encoded local-neighborhood context, relative displacement, and object-distance information. Therefore, this path provides complementary spatial cues for candidate subject–object pair scoring.

In candidate subject–object pairs, spatial distances can vary substantially across different entity pairs. If the same spatial-branch scale is used for all candidate pairs, the GVA-enhanced spatial score cannot be scaled in accordance with pair-level distance variations. Therefore, when fusing the visual pair score and the GVA-enhanced spatial score, we introduce the normalized subject–object distance into a gate function and generate a pair-specific scaling factor for each candidate pair.

For an ordered candidate pair (i,j), we use the previously defined Euclidean distance $d_{ij}$ between their soft centroids for distance-aware gating. Since $p_{i},p_{j}\in {\left[0,1\right]}^{2}$, the maximum distance on the normalized image plane is $\sqrt{2}$. The normalized distance is therefore defined as

(24) $$t_{ij}=\frac{d_{ij}}{\sqrt{2}},\;t_{ij}\in \left[0,1\right].$$

Then, $t_{ij}$ is passed through a two-layer MLP followed by a sigmoid function to obtain the distance-aware gate:

(25) $$\gamma_{ij}=\sigma \left(W_{2}\;\delta \left(W_{1}t_{ij}+b_{1}\right)+b_{2}\right),\;\gamma_{ij}\in \left[0,1\right],$$

where δ(·) denotes the ReLU activation function. In our implementation, the hidden dimension of the MLP is 64, and the bias of the last linear layer is initialized to −2.0, so the spatial branch is introduced conservatively at the beginning of training. The final pair score is computed as

(26) $$S_{ij}^{pair}=S_{ij}^{vis}+\gamma_{ij}S_{ij}^{gva}.$$

In this formulation, $S_{ij}^{vis}$ provides pair-matching information from the visual path, $S_{ij}^{gva}$ provides spatial evidence from local-neighborhood context and relative geometry, and $\gamma_{ij}$ supplies a distance-conditioned spatial scale for the corresponding candidate pair. As a result, $S_{ij}^{pair}$ combines visual information with distance-conditioned spatial information and is subsequently used for matrix refinement and Top-$K_{r}$ candidate pair selection. The gate learns this scaling from normalized subject–object distance, so short-range and long-range pairs can receive different spatial scales without imposing a fixed hand-crafted distance-decay rule.

### 3.7. Matrix Refinement and Top-$K_{r}$ Candidate Selection

After dual-path candidate scoring, SAPPM obtains the full directed candidate-pair score matrix:

(27) $$S^{pair}={\left[S_{ij}^{pair}\right]}_{i,j=1}^{N}\in R^{N\times N}.$$

Here, $S_{ij}^{pair}$ denotes the candidate pair score when object *i* takes the subject role and object *j* takes the object role. This matrix is obtained by fusing the visual score with the distance-modulated spatially enhanced score. As a result, $S^{pair}$ encodes both role-aware visual matching information derived from the original object query features and mask-level spatial context introduced by the spatially enhanced object features.

To obtain pair-importance responses suitable for candidate selection, SAPPM applies the matrix refinement function U(·) to the pair proposal matrix:

(28) $$I=U(S^{pair}).$$

After dual-path candidate-pair estimation, $S^{pair}$ forms a dense pair proposal matrix over the full N×N directed subject–object pair space. Each entry represents the response of an ordered candidate pair, and subsequent Top-$K_{r}$ selection is performed according to this matrix. Therefore, before selecting a fixed number of candidate pairs, U(·) is used to further estimate pair importance and produce the refined importance matrix *I*.

Specifically, U(·) treats $S^{pair}\in R^{B\times N\times N}$ as a single-channel two-dimensional response map. The input matrix is first expanded along the channel dimension:

(29) $$X_{0}=\mathrm{unsqueeze}\left(S^{pair}\right)\in R^{B\times 1\times N\times N}.$$

Then, a three-layer CNN-based matrix learner computes

(30) $$X_{1}=\delta \left({\mathrm{Conv}}_{1\to 64}^{7\times 7}\left(X_{0}\right)\right),$$

(31) $$X_{2}=\delta \left({\mathrm{Conv}}_{64\to 64}^{7\times 7}\left(X_{1}\right)\right),$$

(32) $$I=\mathrm{squeeze}\left({\mathrm{Conv}}_{64\to 1}^{7\times 7}\left(X_{2}\right)\right),$$

where δ(·) denotes the ReLU activation function. All three convolutional layers use stride 1 and padding 3, so the N×N matrix resolution is preserved. The first convolutional layer maps the single-channel pair response map to 64 intermediate channels, the second convolutional layer keeps 64 channels, and the last convolutional layer maps the 64-channel feature map back to a single-channel output. No activation function is applied after the last convolutional layer, so *I* is kept as unnormalized pair-importance logits. This refinement function does not use an additional normalization layer or dropout.

In the overall pipeline, U(·) is applied after dual-path pair proposal estimation and before diagonal suppression and Top-$K_{r}$ selection. During training, *I* is directly supervised by the pair matching loss $L_{match}$. The target matrix is constructed from the assigned ground-truth-directed subject–object pairs, where positive entries correspond to valid ground-truth pairs, and the remaining entries are treated as negative candidates. Thus, the parameters of U(·) are optimized jointly with the pair proposal branch without separate pretraining. During inference, *I* is used as the refined pair-importance matrix for subsequent diagonal masking, flattening, and Top-$K_{r}$ candidate pair selection.

Before Top-$K_{r}$ selection, diagonal entries corresponding to self-pairs are suppressed by setting their scores to −∞ because a valid relation triplet requires two distinct object instances:

(33) $${\hat{I}}_{ij}=\left\{\begin{array}{cc} I_{ij}, & i\neq j,\\ -\infty , & i=j. \end{array}\right.$$

The model then flattens $\hat{I}$ into an $N^{2}$-dimensional vector and selects the top $K_{r}$ entries:

(34) $$\Omega =\mathrm{TopK}\left(\mathrm{Flatten}\left(\hat{I}\right),K_{r}\right).$$

Here, Ω denotes the selected flattened pair indices. We set $K_{r}=100$, matching the number of relation queries in our implementation. Under one-based indexing, for each selected flattened index ω∈Ω, the corresponding subject index and object index are recovered as

(35) $$u=\left\lfloor \frac{\omega -1}{N}\right\rfloor +1,\;v=\left(\omega -1\right)\;\mathrm{mod}\;N+1.$$

The final selected pairs are then obtained as

(36) $$P={\left\{\left(u_{k},v_{k}\right)\right\}}_{k=1}^{K_{r}}.$$

Top-$K_{r}$ candidate selection converts the full N×N directed subject–object pair space into a fixed number of selected pairs. The relation decoder operates only on these selected pairs; therefore, this selection step determines the candidate scope for relation decoding.

### 3.8. Relation Decoding and Triplet Prediction

After Top-$K_{r}$ selection, SAPPM obtains a set of directed subject–object pairs for predicate category prediction. The pair proposal matrix determines which entity pairs are retained for subsequent prediction, but the predicate category of each retained pair still needs to be inferred. Therefore, the relation decoder learns relation representations on the selected pairs and converts them into final subject–predicate–object triplet predictions.

For the *k*-th selected pair $\left(u_{k},v_{k}\right)$, we obtain the corresponding subject feature $q_{u_{k}}$ and object feature $q_{v_{k}}$ from the object queries, and apply two linear transformations:

(37) $${\hat{q}}_{u_{k}}=W_{s}q_{u_{k}},\;{\hat{q}}_{v_{k}}=W_{o}q_{v_{k}}.$$

Here, $W_{s}$ and $W_{o}$ distinguish the subject side and the object side in a directed relation. The subject-side features and object-side features of all selected pairs are then concatenated along the sequence dimension to form the pair-level memory:

(38) $$Q^{pair}={\mathrm{Concat}}_{\mathrm{seq}}\left({\left\{{\hat{q}}_{u_{k}}\right\}}_{k=1}^{K_{r}},{\left\{{\hat{q}}_{v_{k}}\right\}}_{k=1}^{K_{r}}\right),$$

where $Q^{pair}\in R^{2K_{r}\times B\times C}$. This representation preserves the directed structure of the selected pairs, allowing predicate prediction to use the order information between subject and object.

The relation decoder uses $K_{r}$ learnable relation queries $Q^{rel}\in R^{K_{r}\times B\times C}$. These queries interact with the pair-level memory and produce relation representations for the selected pairs:

(39) $$R=\mathrm{Decoder}(Q^{rel},Q^{pair}).$$

For the *k*-th selected pair, the output representation $R_{k}$ is passed through a linear classifier to obtain predicate logits:

(40) $$z_{k}^{rel}=W_{rel}R_{k},$$

and the predicate category probability is computed as

(41) $$p_{k}^{r}=\mathrm{Softmax}\left(z_{k}^{rel}\right).$$

Together with the subject/object category predictions and mask predictions from the object parsing branch, each selected pair forms a subject–predicate–object triplet.

The relation decoder consists of six Transformer decoder layers. Each layer contains cross-attention between relation queries and the pair-level memory, self-attention among relation queries, and a feed-forward network. Layer normalization is applied after the attention and feed-forward operations. The number of attention heads is 8, the feature dimension is C=256, and the hidden dimension of the feed-forward network is 2048 with ReLU activation. The FFN dropout is set to 0.1, while the attention dropout and projection dropout are both set to 0.0.

During training, the Top-$K_{r}$ candidate predictions are first matched to ground-truth triplets. Let the *t*-th ground-truth triplet be $\left(s_{t},r_{t},o_{t}\right)$, where $r_{t}$ denotes the predicate category, and let $c_{t}^{s}$ and $c_{t}^{o}$ denote the subject category and object category, respectively. For candidate prediction *k*, the subject category probability and object category probability are denoted as $p_{k}^{s}$ and $p_{k}^{o}$. The Hungarian matching cost is defined as

(42) $$C_{k,t}=-\log p_{k}^{s}\left(c_{t}^{s}\right)-\log p_{k}^{o}\left(c_{t}^{o}\right).$$

The predicate classification cost has zero weight in this matching stage, so the matching is based on the consistency between candidate predictions and ground-truth triplets in subject/object categories.

Let M denote the matched ground-truth triplet set obtained by Hungarian matching, and let σ(t) denote the candidate prediction matched to the *t*-th ground-truth triplet. For matched candidate predictions, the classification losses are defined as

(43) $$L_{s\text{-}cls}=-\frac{1}{\left|M\right|}\sum_{t\in M}\log p_{\sigma (t)}^{s}\left(c_{t}^{s}\right),$$

(44) $$L_{o\text{-}cls}=-\frac{1}{\left|M\right|}\sum_{t\in M}\log p_{\sigma (t)}^{o}\left(c_{t}^{o}\right),$$

(45) $$L_{r\text{-}cls}=-\frac{1}{\left|M\right|}\sum_{t\in M}\log p_{\sigma (t)}^{r}\left(r_{t}\right).$$

Here, $L_{s\text{-}cls}$, $L_{o\text{-}cls}$, and $L_{r\text{-}cls}$ are the cross-entropy losses for subject category, object category, and predicate category, respectively. Unmatched candidate predictions do not contribute to these three classification losses. The three losses form the relation-side classification objective:

(46) $$L_{cls}=\lambda_{s}L_{s\text{-}cls}+\lambda_{o}L_{o\text{-}cls}+\lambda_{r}L_{r\text{-}cls},$$

where $\lambda_{s}$, $\lambda_{o}$, and $\lambda_{r}$ denote the corresponding loss weights. This classification objective corresponds to learning the subject, object, and predicate categories of matched triplets, while the pair matching loss $L_{match}$ acts on the pair proposal matrix to learn the ranking of ground-truth directed subject–object pairs among candidate pairs. Through these two types of losses, candidate-pair selection and subsequent triplet classification are kept consistent during training.

## 4. Experiments

### 4.1. Dataset and Evaluation Protocol

We evaluate the proposed method on the Panoptic Scene Graph (PSG) benchmark [8]. The PSG dataset is built upon COCO images and their panoptic segmentation annotations, and further provides predicate annotations between objects. It contains 133 object categories and 56 predicate categories. Different from conventional box-level scene graph generation, PSG represents both foreground objects and background regions with panoptic masks, and requires the model to predict subject–predicate–object triplets based on pixel-level object parsing. Therefore, this task provides a direct setting for evaluating the effect of mask-level object representations on visual relation modeling.

Following the standard Scene Graph Detection (SGDet) evaluation protocol, the model is required to jointly predict object categories, object masks, and predicate relations between objects. A predicted triplet is considered correctly recalled only when the predicted subject and object masks both achieve an IoU of at least 0.5 with their corresponding ground-truth objects, and the subject category, predicate category, and object category are all correctly predicted. Therefore, SGDet performance is jointly affected by object parsing quality, candidate subject–object pair selection, and predicate classification.

We report R@20, R@50, R@100 and mR@20, mR@50, mR@100 as the main evaluation metrics. R@K measures the overall triplet recall among the top-*K* confidence-ranked predicted triplets, whereas mR@K first computes recall for each predicate category and then averages the results over all categories, reducing the dominance of frequent predicate classes. Since the predicate distribution in PSG is highly imbalanced, both R@K and mR@K are reported to reflect overall triplet recall and category-balanced recall, respectively.

In addition to triplet-level SGDet metrics, we further report proposal-level pair recall as an auxiliary metric for analyzing the retention ability of candidate subject–object pairs. This metric only examines whether a ground-truth directed subject–object pair is retained in the Top-*K* pair proposals, without requiring correct predicate classification. It can therefore provide a relatively independent measure of the candidate coverage quality at the pair proposal stage.

### 4.2. Implementation Details

The model adopts ResNet-50 as the backbone network, and the object parsing branch is initialized with COCO-pretrained Mask2Former weights. We train the model in a single-stage setting with the Mask2Former-based object parsing branch frozen. In all main experiments, we use 100 object queries and 100 relation queries, and set the channel dimension of object query features to 256. The relation decoder uses six Transformer decoder layers with 8 attention heads, an embedding dimension of 256, an FFN hidden dimension of 2048, and an FFN dropout rate of 0.1; no dropout is applied to the attention weights or output projections. In the SAPPM configuration used in the main experiments, the neighborhood size $K_{n}$ of the 2D KNN module is set to 4. Since the center object query is included in the KNN set, each object query aggregates information from itself and at most three nearby object queries. The number of channel groups *G* in GVA is set to 8. With the object-query feature dimension C=256, each group contains 32 channels. These settings are used unless otherwise specified. During training, multi-scale resize augmentation is applied, where the image scale is randomly sampled from 480 to 800 pixels while preserving the aspect ratio. During testing, single-scale input is used with an image size of 1333×800. During training, the frozen object parsing branch provides object query features, category predictions, and mask predictions, while the relation decoder performs predicate prediction based on the selected pairs. Accordingly, only the pair proposal module, subject/object role projections, the relation decoder, and the predicate classification head are optimized.

In all experiments, the matrix refinement function U(·) is trained as part of the SAPPM pair proposal branch. It takes the pair proposal matrix $S^{pair}$ as input, produces the refined importance matrix *I*, and is used before diagonal suppression and Top-$K_{r}$ candidate pair selection. U(·) is not separately pretrained; its parameters are optimized together with the other relation-side parameters using AdamW and are directly supervised by the pair matching loss $L_{match}$.

Under this setting, the total training loss is defined as

(47) $$L=L_{r\text{-}cls}+L_{s\text{-}cls}+L_{o\text{-}cls}+L_{match}.$$

Here, $L_{r\text{-}cls}$ is the predicate classification loss for relation-query outputs that are matched to ground-truth triplets. $L_{s\text{-}cls}$ and $L_{o\text{-}cls}$ are cross-entropy losses on the subject and object category predictions of the matched selected pairs, respectively. The selected pairs are first obtained by applying Top-$K_{r}$ selection to the refined pair proposal matrix. They are then matched to ground-truth triplets using Hungarian matching. The matching cost is computed from the predicted subject and object class scores, while the predicate classification cost has zero weight in this matching step. Consequently, only matched selected predictions contribute to the subject, object, and predicate classification losses, whereas unmatched selected predictions are ignored by these three classification terms.

$L_{match}$ supervises the pair proposal matrix before Top-$K_{r}$ selection. To build its target, the Mask2Former-style object-query assignment first maps each ground-truth instance to an object query. Each ground-truth triplet then marks the directed matrix entry from the assigned subject query to the assigned object query as positive, and the remaining directed entries are treated as negative. In the SAPPM setting, $L_{match}$ is implemented as binary focal BCE over this pair-presence matrix, with targets binarized as 1[gt_importance>0]. This formulation trains the refined importance logits *I* to rank true directed subject–object pairs above non-matching pairs while reducing the effect of the extreme positive–negative imbalance in the full N×N pair space. The loss weights for $L_{s\text{-}cls}$, $L_{o\text{-}cls}$, and $L_{r\text{-}cls}$ are set to 4.0, 4.0, and 2.0, respectively.

The trainable parameters are optimized using AdamW. Specifically, the optimizer is applied to the relation-side modules, including the SAPPM pair proposal module, subject/object role projections, relation queries, relation decoder, and predicate classification head, while the Mask2Former-based object parsing branch remains frozen during training. The initial learning rate and weight decay are both set to $1\times 10^{-4}$. To improve training stability, gradient clipping is applied with a maximum norm of 0.1 under the $L_{2}$ norm. An epoch-based step decay learning-rate schedule is adopted. The learning rate is decayed at the 5th and 10th epochs with a decay factor of γ=0.5. Therefore, the learning rate is $1\times 10^{-4}$ for epochs 1–5, $5\times 10^{-5}$ for epochs 6–10, and $2.5\times 10^{-5}$ for epochs 11–14. The reported results are obtained after 14 training epochs. All experiments are conducted on a computing server running Ubuntu 24.04 and equipped with an Intel^®^ Xeon^®^ Platinum 8570 Processor CPU with a base frequency of 2.1 GHz and a maximum turbo frequency of 4.0 GHz, 128 GB DDR5 RAM, and two NVIDIA GeForce RTX 4090 GPUs. The computing server was manufactured by Dongguan Yunjiyigao Information Technology Co., Ltd. (Dongguan, Guangdong, China). During training, 4 images are processed on each GPU, resulting in a total batch size of 8. Unless otherwise specified, all experiments follow the same optimization and training settings.

### 4.3. SGDet Main Results

This section reports SGDet results on the PSG benchmark together with representative published PSG methods. Table 1 summarizes R@20/50/100, mR@20/50/100, and the six-metric average. These results provide an overview of how SAPPM performs in relation to existing PSG methods under the commonly used SGDet evaluation protocol. To further isolate the effect of SAPPM, we also provide controlled comparisons and ablation studies under the same training setting in the following sections.

As shown in Table 1, SAPPM achieves competitive performance across the SGDet evaluation metrics. It obtains 32.5, 37.7, and 40.7 on R@20, R@50, and R@100, respectively, and reaches 27.4, 29.9, and 31.9 on mR@20, mR@50, and mR@100, respectively. These results indicate that SAPPM remains competitive under the SGDet benchmark, while the component-level contribution of the proposed pair proposal design is examined through controlled experiments in the following sections.

In particular, the R@20 and mR@20 results suggest that SAPPM produces competitive top-ranked triplet predictions, where candidate-pair ranking is especially important under the limited Top-$K_{r}$ evaluation setting. The subsequent controlled comparisons further verify that incorporating mask-level spatial cues into pair proposal improves candidate subject–object pair selection and benefits relation prediction.

### 4.4. Model Scale, Training Overhead, Inference Efficiency, and Spatial-Path Complexity

To assess the computational cost of SAPPM, we report model scale, wall-clock training time, end-to-end inference efficiency, and the estimated complexity of the spatial path. The results are summarized in Table 2.

As shown in Table 2a, the baseline contains 54.28 M parameters, whereas SAPPM contains 54.88 M parameters. Thus, the proposed spatially aware pair proposal module introduces 0.60 M additional parameters, corresponding to approximately 1.10% of the baseline model size. In terms of wall-clock training time, the baseline requires 6.16 h for 14 epochs, while SAPPM requires 6.67 h under the same training setting, leading to an additional training time of 0.51 h. These results indicate that SAPPM adds only a small amount of model scale and training cost.

Table 2b further reports the end-to-end inference efficiency under two object-query settings. When N=100, SAPPM increases latency from 96.36 ms to 99.39 ms and changes FPS from 10.38 to 10.06. When N=200, latency increases from 109.02 ms to 110.81 ms, and FPS changes from 9.17 to 9.02. Peak GPU memory is nearly unchanged in both settings. These results show that the practical inference overhead of SAPPM remains small for the tested object-query numbers.

Table 2c gives the estimated computation of the SAPPM spatial path. With fixed neighborhood size $K_{n}$ and feature dimension *C*, the GVA aggregation cost grows mainly with the number of object queries, while the KNN distance computation and dense pair-level scoring involve all candidate subject–object pairs and therefore contain dominant $O(N^{2})$ terms. The estimated spatial-path computation increases from 4.387 GFLOPs at N=100 to 17.079 GFLOPs at N=200, corresponding to about a 3.89× increase. This trend is consistent with the quadratic pair-level operations in SAPPM, while the measured end-to-end latency in panel (b) remains close to the baseline.

### 4.5. Effect of Spatial Structure Modeling on Candidate Pairing

To assess the role of spatial structure in candidate subject–object pair selection, we conduct a component ablation on the visual path, the GVA-enhanced spatial path, and the distance-aware gate. The purpose of this experiment is not to show that spatial cues can replace object-feature-based pairing. Instead, it examines whether spatial cues can provide complementary evidence for pair proposal scoring and improve the retention of ground-truth subject–object pairs within the limited Top-$K_{r}$ candidate set.

All variants are evaluated under the same object parsing branch, Top-$K_{r}$ candidate capacity, number of selected pairs, relation decoder architecture, and SGDet evaluation protocol. The main difference lies in the scoring source of the pair proposal matrix before relation decoding. In the visual path only, pair scores are computed from original object query features after subject/object role projections. This path can exploit object categories, appearance representations, and contextual co-occurrence, but it has limited explicit modeling of relative positions, object distances, and local layout structures. In spatial path only (GVA-enhanced), pair scores are computed from spatially enhanced object features. These features are obtained from mask-level soft centroids, relative position encoding, and GVA-based local-neighborhood aggregation. We compare four proposal forms: visual path only, spatial path only (GVA-enhanced), visual + spatial path, and visual + spatial path + gate. The results are shown in Table 3.

Compared with visual path only, spatial path only (GVA-enhanced) improves all R@K and mR@K metrics. R@20, R@50, and R@100 increase from 30.50, 36.32, and 39.64 to 32.08, 37.30, and 40.23, respectively. mR@20, mR@50, and mR@100 increase from 24.49, 27.70, and 29.62 to 25.92, 28.44, and 29.98, respectively. Because the relation decoder and the number of selected pairs are kept unchanged, these results suggest that introducing relative geometry and local-neighborhood context into pair proposal scoring is associated with better candidate retention. However, the gains on mR@50 and mR@100 are relatively moderate. This indicates that spatially enhanced features alone may not be sufficient for the diverse relation types in PSG. Many functional, action-related, or interaction-oriented relations still depend on object categories, appearance, action semantics, and contextual co-occurrence. Therefore, the GVA-enhanced spatial path should be interpreted as a complementary scoring source rather than a replacement for the visual path.

Directly fusing the visual path and the GVA-enhanced spatial path brings partial gains, but the changes are not consistent across all metrics. Compared with spatial path only (GVA-enhanced), visual + spatial path slightly improves R@20, mR@20, and mR@100, while showing small decreases in R@50, R@100, and mR@50. This suggests that unregulated fusion may not benefit all candidate object pairs in a stable manner. A simple summation of the spatially enhanced score and the visual score may be affected by the different degrees to which candidate pairs depend on spatial cues.

With the distance-aware gate, visual + spatial path + gate obtains the best results in this ablation setting. Compared with visual + spatial path, R@20, R@50, and R@100 increase from 32.20, 37.27, and 40.15 to 32.53, 37.67, and 40.65, respectively. mR@20, mR@50, and mR@100 increase from 26.05, 28.41, and 30.12 to 27.36, 29.86, and 31.89, respectively. The larger gains in mR@K suggest improved category-balanced relation prediction. These results are consistent with the design of the distance-aware gate: it modulates the contribution of the GVA-enhanced spatial path according to the spatial distance between candidate objects. In this way, spatial information is introduced into pair proposal scoring more selectively, rather than being applied to all candidate pairs with a fixed strength.

### 4.6. Effect of Grouped Vector Attention (GVA) on Pair Proposal Ranking

To further examine whether SAPPM brings clearer gains for relations with explicit spatial layout, we define a conservative set of representative spatial-relation diagnostic predicates. This set is used only for analysis and is not used for model training, loss computation, or hyperparameter selection. A predicate is included only when its label meaning is mainly determined by relatively static subject–object geometry that can be described by pair-level spatial cues, including relative location, containment, contact, support, surface layout, or vehicle–road layout. Applying this rule gives 12 included predicates: in front of, beside, in, on, on back of, standing on, sitting on, lying on, leaning on, walking on, parked on, and driving on. Table A1 in Appendix A gives the explicit selection and exclusion criteria, and Table A2 reports the number of validation instances and predicate-level Rec@100 for each included predicate.

**Figure 3:**
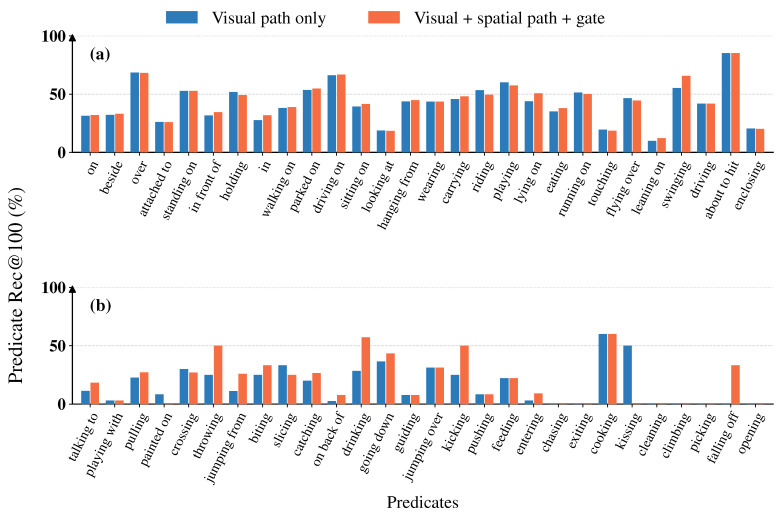
Predicate-level Rec@100 comparison across all 56 predicates: (**a**) the first group of predicates; (**b**) the second group of predicates. Baseline denotes the visual-path-only model, and SAPPM denotes the full model with spatially aware pair proposal. An invisible bar indicates that the corresponding Rec@100 value is 0, rather than missing data.

The remaining 44 PSG predicate categories are not treated as representative spatial-relation diagnostic predicates. This choice follows the same conservative, semantics-driven criterion. Some predicates contain spatial words, but their labels are governed mainly by motion state, pose, viewpoint, temporal trajectory, or local appearance rather than by stable pair-level layout. For example, painted on, running on, flying over, and crossing are not included in the representative set: painted on depends strongly on local surface appearance and texture correspondence; running on and crossing depend on action state, pose, and motion path; and flying over is affected by depth, viewpoint, and large-scale scene layout. For the same reason, falling off is not used as a representative static spatial-relation predicate: it describes a transition away from a support surface, so its recognition requires action-state evidence in addition to pair geometry. Predicates such as wearing and holding are also excluded because their recognition is dominated by object semantics, affordance, and human–object interaction cues. These excluded predicates are still evaluated in the all-56-predicate analysis in Figure 3.

**Figure 4:**
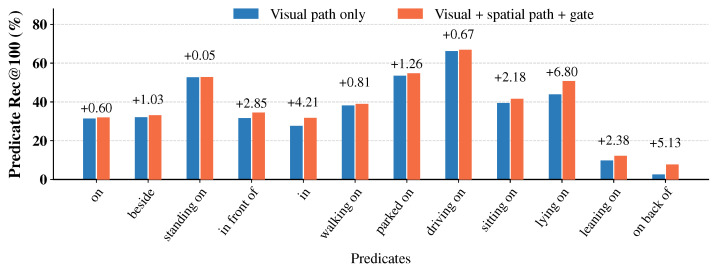
Comparison of predicate-level Rec@100 for the 12 representative spatial-relation diagnostic predicates.

Based on the 12 representative spatial-relation predicates, we compare visual path only and SAPPM using predicate-level Rec@100. Figure 4 visualizes these 12 predicates for readability, while the corresponding numerical values and sample counts are provided in Table A2. This analysis characterizes trends for predicates whose semantics are directly tied to static spatial layout, while the broader predicate-wise behavior is examined separately in the all-56-predicate comparison.

We further compare visual path only and SAPPM over all 56 predicate categories, as shown in Figure 3. This analysis is needed because SAPPM changes the ranking of selected subject–object pairs before relation decoding, rather than directly changing the predicate classifier. In the implementation, the final pair score is computed from the visual pair score and the gated GVA-based spatial score, and the Top-$K_{r}$ pairs are selected from the refined N×N pair matrix. Therefore, a predicate-level Rec@100 value can increase when spatial cues help its ground-truth pairs enter the selected pair set, but it can also decrease when the new ranking gives higher positions to other competing pairs.

For the 12 representative spatial-relation predicates in Table A2, SAPPM improves every listed predicate. This behavior is expected because these predicates are mainly defined by a stable pair-level layout, such as relative position, containment, support, contact, or road-surface association. For these categories, mask centroids, local neighborhoods, and relative geometry provide useful evidence for ranking the correct subject–object pair higher before the relation decoder.

The all-56-predicate comparison also shows that the gains vary across predicate types. This is consistent with the ranking role of SAPPM. Since Top-$K_{r}$ selection has a fixed capacity, increasing the rank of layout-supported pairs can change the relative positions of other candidate pairs. The few decreases mainly occur for predicates whose labels depend less on static pair geometry and more on appearance, action state, pose, motion path, depth, or viewpoint. For example, painted on depends on local surface appearance and texture correspondence; running on and crossing depend on action state and motion path; and flying over depends on depth ordering, viewpoint, and broader scene context. For these predicates, centroid-level geometry is useful as complementary ranking evidence, but the final label is also shaped by visual and semantic cues beyond pair-level layout.

The predicates wearing and holding further show that the effect of SAPPM is not identical across predicate types. The soft centroid, relative geometry, and local-neighborhood layout introduced by SAPPM mainly affect candidate-pair ranking before relation decoding, helping the model retain more suitable subject–object pairs within the limited Top-$K_{r}$ candidate set. For relations that depend more on pair-level spatial configuration, these mask-derived spatial cues can provide complementary information for candidate ranking. However, the predicate categories of wearing and holding are usually not determined only by the overall spatial positions of the subject and object. Their recognition is also related to object semantics, category compatibility, local human pose, hand–object contact, attachment patterns, object affordance, and fine-grained appearance evidence. Therefore, these interaction-dominant predicates have a more complex dependence on pair-level spatial cues, and centroid-level geometry and local layout are difficult to fully represent the local interaction information required for their discrimination.

In the predicate-level Rec@100 comparison over all 56 predicate categories, wearing remains nearly unchanged, changing from 43.50% to 43.65%, while holding decreases slightly from 51.88% to 49.13%. This result indicates that SAPPM does not cause a uniform degradation on these interaction-dominant predicates. At the same time, the decrease on holding suggests that when a predicate depends more strongly on hand–object contact, human pose, object affordance, and local appearance details, spatial ranking information based on soft centroids, relative geometry, and local-neighborhood layout may not cover all fine-grained cues required by the predicate. Overall, the main benefit of SAPPM comes from candidate-pair ranking before relation decoding, especially for relations that rely more on pair-level spatial configuration. For predicates such as wearing and holding, visual information, category cues, and fine-grained interaction evidence remain important factors.

Overall, Table 3, Figure 3 and Figure 4, and Appendix A show that GVA provides complementary spatial cues for pair proposal scoring. The main effect is to change the candidate-pair ranking before relation decoding. This helps predicates whose labels are directly tied to static spatial layout, while predicates dominated by action, appearance, affordance, or fine-grained local interaction may show smaller gains or slight decreases.

### 4.7. Comparison with Simplified Spatial Proposal Variants

To further assess the contribution of GVA in the spatially aware pair proposal, we compare SAPPM with two controlled alternatives while keeping the rest of the framework unchanged. Visual + Geo-MLP keeps the visual scoring branch and the same distance-aware gate, but replaces the GVA-RPE branch with a three-layer geometry MLP. The MLP takes six pairwise geometry features as input: Δx, Δy, |Δx|, |Δy|, Euclidean distance, and squared distance. It does not use KNN neighborhood construction, relative position encoding, or neighbor-feature aggregation. Visual + RPE-only keeps the KNN neighborhood construction, neighboring value features, and relative position encoding, but removes grouped vector attention. Instead, it simply averages the neighboring value features and their RPE embeddings before pair scoring. For all settings, the detector, relation decoder, number of selected pairs, matrix refinement module, training schedule, and SGDet evaluation protocol are kept unchanged. Only the pair proposal module used for selected pair ranking is modified.

The comparison results are shown in Table 4. The results show that both controlled alternatives improve the visual-only proposal in most metrics, confirming that explicit spatial cues are useful for pair proposal ranking. However, the two simplified variants have different limitations. Visual + Geo-MLP improves R@K, but its mR@K gains are limited, and mR@100 is slightly lower than the visual-only baseline. This suggests that direct pairwise geometry helps recover some common spatial patterns, but is less effective for balanced predicate coverage. Visual + RPE-only improves all six metrics over the visual-only baseline, showing that KNN-based neighborhood context and relative position encoding are more informative than pairwise geometry alone. Nevertheless, SAPPM achieves the best performance across all six metrics. Compared with RPE-only, the gains indicate that grouped vector attention is useful because it learns adaptive, channel-group-wise weights over local neighbors, rather than treating all neighboring cues with the same mean aggregation.

### 4.8. Effect of Spatial Anchor Choice

To evaluate only the effect of the spatial-anchor choice, all variants in this ablation are evaluated under the same controlled setting. Specifically, the object parsing branch, SAPPM structure, GVA module, distance-aware gate, relation decoder, training schedule, number of object and relation queries, Top-$K_{r}$ candidate capacity, and SGDet evaluation protocol are kept unchanged. The only difference lies in how the object spatial anchor $p_{i}$ is computed before KNN construction, relative position encoding, and distance-aware pair scoring. Bounding-box center first thresholds the predicted mask probability at 0.5, obtains the tight foreground bounding box, and uses the box center as the anchor. Binary mask centroid uses the same thresholded foreground mask but averages the foreground pixel coordinates. Sigmoid-based soft centroid uses sigmoid mask probabilities as coordinate weights. ReLU-logit soft centroid uses only positive mask logits as coordinate weights, preserving their relative response magnitudes while suppressing non-positive regions.

As shown in Table 5, the proposed ReLU-logit soft centroid achieves the best performance among the compared spatial anchor choices, obtaining 32.53/37.67/40.65 on R@20/R@50/R@100 and 27.36/29.86/31.89 on mR@20/mR@50/mR@100. Compared with the sigmoid-based soft centroid, the improvements on R@K are moderate, with gains of 0.18, 0.20, and 0.24 points at R@20, R@50, and R@100, respectively. By contrast, the improvements on mR@K are more pronounced, reaching 1.52, 1.34, and 1.69 points. Since all variants keep the same relation decoder and differ only in how the spatial anchor is computed, this difference is mainly attributed to changes in candidate-pair ranking before relation decoding. The larger margins under category-averaged evaluation indicate that the ReLU-logit centroid is particularly useful for relation categories whose proposal scores are more sensitive to relative geometry and local spatial layout.

This result is consistent with the way spatial anchors are used in SAPPM. The estimated centroids determine the 2D KNN neighborhoods, the relative position encodings, and the pairwise distances used by the distance-aware gate. Therefore, the quality of the anchor affects not only the object position itself, but also the subsequent local aggregation and pair-level spatial modulation. The sigmoid-based centroid provides a smooth estimate, but low-confidence regions still contribute nonzero weights after the probability transformation, and large positive logits are compressed into a saturated range. The binary mask centroid suppresses low-confidence regions through hard thresholding, but also removes response-magnitude differences within the predicted mask. The bounding-box center serves as a coarse spatial reference and remains competitive on several R@K metrics, but its lower mR@K scores suggest that rectangular box centers are less suitable for balanced relation coverage when fine-grained mask-level geometry is needed. In comparison, the ReLU-logit centroid retains only positive mask responses while preserving their relative magnitudes, thereby providing a more discriminative spatial anchor for KNN construction, relative geometry encoding, and distance-aware pair scoring.

### 4.9. Pair Proposal Analysis

To further analyze whether the performance gain of SAPPM is related to improved candidate-pair retention, we report Proposal Recall@K (PR@K) and mean Proposal Recall@K (mPR@K). These are proposal-level metrics. They are computed before relation decoding and do not evaluate whether predicate classification is correct. PR@K measures whether the ground-truth directed subject–object pair of a relation is retained in the Top-*K* pair proposals. mPR@K further averages this proposal recall over predicate categories. Thus, PR@K and mPR@K evaluate pair proposal quality, whereas R@K and mR@K evaluate final triplet-level relation prediction.

Let the evaluation set contain *N* images. For the *n*-th image, the set of ground-truth relations is defined as

(48) $$G^{\left(n\right)}={\left\{\left(s_{t},o_{t},r_{t}\right)\right\}}_{t=1}^{T_{n}},$$

where $s_{t}$ and $o_{t}$ denote the subject and object instance indices of the *t*-th ground-truth relation, and $r_{t}$ denotes its predicate category. According to the pair proposal matrix, the model selects the Top-*K* directed candidate pairs:

(49) $$P_{K}^{\left(n\right)}={\left\{\left(i_{k},j_{k}\right)\right\}}_{k=1}^{K}.$$

For a ground-truth relation instance $\left(s_{t},o_{t},r_{t}\right)\in G^{\left(n\right)}$, the corresponding directed subject–object pair is regarded as retained if there exists a predicted pair $\left(i_{k},j_{k}\right)\in P_{K}^{\left(n\right)}$ whose subject and object masks match the ground-truth subject and object masks, respectively, under an IoU threshold of 0.5. The coverage indicator is defined as

(50) $$\begin{array}{c} \delta_{K}^{\left(n\right)}\left(s_{t},o_{t}\right)=I[\exists \left(i_{k},j_{k}\right)\in P_{K}^{\left(n\right)}:\mathrm{IoU}\left(M_{i_{k}}^{\mathrm{pred},(n)},M_{s_{t}}^{\mathrm{gt},(n)}\right)\geq 0.5\\ \wedge \;\mathrm{IoU}\left(M_{j_{k}}^{\mathrm{pred},(n)},M_{o_{t}}^{\mathrm{gt},(n)}\right)\geq 0.5]. \end{array}$$

This definition enforces directed pair matching. Only the correct direction $\left(s_{t},o_{t}\right)$ is counted as retained; the reversed pair $\left(o_{t},s_{t}\right)$ is not equivalent.

The dataset-level PR@K is computed as

(51) $$\mathrm{PR}@K=\frac{\sum_{n=1}^{N}\sum_{\left(s_{t},o_{t},r_{t}\right)\in G^{\left(n\right)}}\delta_{K}^{\left(n\right)}\left(s_{t},o_{t}\right)}{\sum_{n=1}^{N}\left|G^{\left(n\right)}\right|}.$$

To evaluate proposal retention in a predicate-category-balanced manner, we further compute predicate-wise pair recall. For predicate category *c*, the subset of ground-truth relations in the *n*-th image is

(52) $$G_{c}^{\left(n\right)}=\left\{\left(s_{t},o_{t},r_{t}\right)\in G^{\left(n\right)}|r_{t}=c\right\}.$$

The proposal recall for predicate category *c* is defined as

(53) $${\mathrm{PR}}_{c}@K=\frac{\sum_{n=1}^{N}\sum_{\left(s_{t},o_{t},r_{t}\right)\in G_{c}^{\left(n\right)}}\delta_{K}^{\left(n\right)}\left(s_{t},o_{t}\right)}{\sum_{n=1}^{N}\left|G_{c}^{\left(n\right)}\right|}.$$

Let $C_{\mathrm{valid}}=\{c|\sum_{n=1}^{N}|G_{c}^{\left(n\right)}\left|>0\right\}$ denote the set of predicate categories with ground-truth samples in the evaluation set. Then mPR@K is defined as

(54) $$\mathrm{mPR}@K=\frac{1}{|C_{\mathrm{valid}}|}\sum_{c\in C_{\mathrm{valid}}}{\mathrm{PR}}_{c}@K.$$

Therefore, mPR@K is a predicate-category-balanced proposal-level metric. It should not be interpreted as object-category-balanced recall or as final triplet-level recall.

Pair-level recall is not sufficient for final SGDet performance, because retained candidate pairs still require correct predicate classification. Nevertheless, in a pair proposal–relation decoding pipeline, a ground-truth subject–object pair excluded from the Top-$K_{r}$ candidate set cannot be processed by the relation decoder. Proposal-level recall therefore provides a useful diagnostic view of candidate retention before relation prediction. Table 6 compares Pair/Proposal R@20 and Triplet R@20 under the same evaluation protocol.

Table 7 reports the overall pair proposal quality. SAPPM improves PR@20, PR@50, and PR@100 from 57.48, 69.44, and 75.76 to 60.46, 71.15, and 76.51, respectively. It also improves mPR@20, mPR@50, and mPR@100 from 67.34, 76.71, and 80.65 to 69.90, 77.35, and 81.16, respectively. The gains are larger under smaller candidate capacities. PR@20 and mPR@20 increase by 2.98 and 2.56 points, whereas PR@100 and mPR@100 increase by 0.75 and 0.51 points. This pattern suggests that SAPPM mainly improves the ranking quality of top-ranked proposals, rather than only increasing marginal coverage when the candidate set is already large.

We further analyze pair proposal recall under different subject–object distance ranges. This analysis groups GT relation instances according to the spatial distance between the GT subject mask and the GT object mask. It is designed to examine whether a spatially aware pair proposal is associated with better retention of ground-truth directed subject–object pairs under different distance conditions. For distant subject–object pairs, direct contact cues are often weak or absent. In such cases, object categories, appearance representations, and semantic co-occurrence may be insufficient for stable top-ranked proposal scoring. Relative position, global layout, and contextual consistency may therefore provide useful complementary evidence.

Let the GT mask centroids of the subject and object be $c_{s}=\left(x_{s},y_{s}\right)$ and $c_{o}=\left(x_{o},y_{o}\right)$, respectively. Let the image width and height be *W* and *H*. The normalized distance is defined as

(55) $$d\left(s,o\right)=\frac{\sqrt{{\left(x_{s}-x_{o}\right)}^{2}+{\left(y_{s}-y_{o}\right)}^{2}}}{\sqrt{W^{2}+H^{2}}}.$$

According to d(s,o), all GT relation instances are divided into three groups:

(56) Near:d<0.3,Mid:0.3≤d<0.6,Far:d≥0.6.

The distance bins are computed from GT mask centroids rather than predicted masks. This avoids making the grouping itself depend on model-specific mask prediction errors. The results are shown in Table 8.

SAPPM improves PR@K in the Near, Mid, and Far groups. The gains are largest in the Far group, where PR@20, PR@50, and PR@100 increase by 10.34, 13.80, and 8.62 points, respectively. This result should be interpreted at the proposal stage: after grouping relation instances by GT mask-centroid distance, distant ground-truth subject–object pairs are retained more often in the Top-*K* proposals. The result suggests that relative geometry and local-neighborhood context provide useful ranking evidence when direct visual contact is weak. This distance-binned result does not conflict with the mixed predicate-level trends in Figure 3, because PR@K measures candidate-pair retention before relation decoding, whereas predicate-level Rec@100 also depends on the predicate classifier and category-specific visual or semantic cues.

Overall, the proposal-level analysis supports the interpretation that SAPPM improves candidate retention before relation decoding. The improvements in PR@K, mPR@K, and distance-binned PR@K are consistent with the final SGDet gains, but they should be interpreted as proposal-level evidence rather than final predicate classification evidence. These results indicate that the benefits of SAPPM are related to better retention and ranking of ground-truth-directed subject–object pairs, especially under limited candidate capacity and for distant object pairs.

### 4.10. Qualitative Visualization Analysis

**Figure 5:**
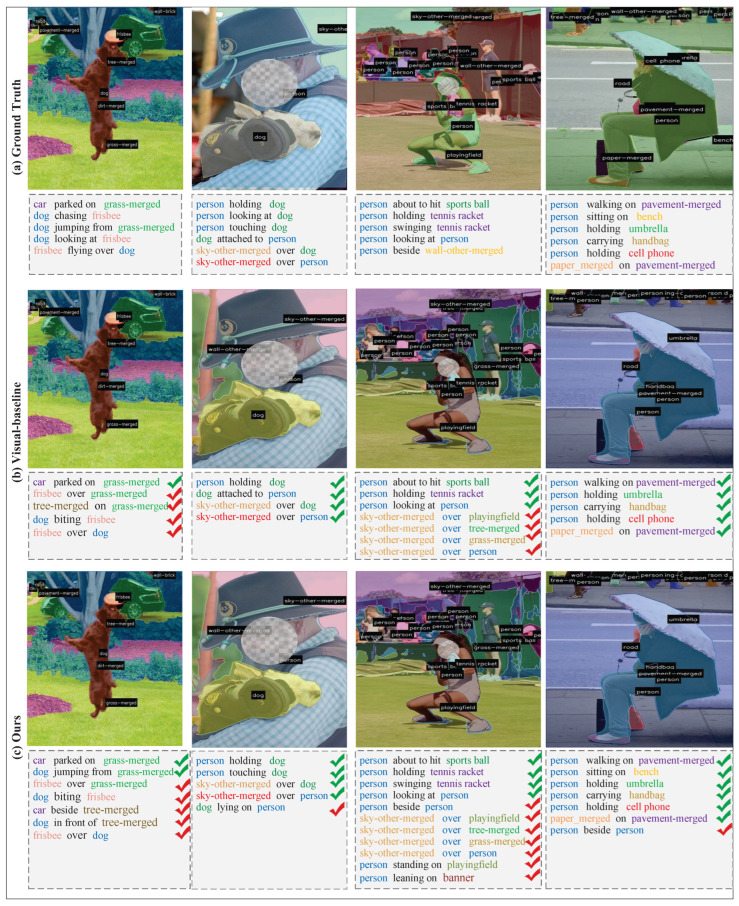
Qualitative visualization results comparing the ground-truth annotations, the predictions of the visual-only baseline, and the predictions of SAPPM. Green check marks denote correct predictions that match the PSG ground-truth annotations and are counted in the quantitative evaluation. Red check marks denote visually plausible relations predicted by SAPPM but not annotated in the PSG ground truth; these relations are shown only for qualitative analysis and are not treated as true positives under the official evaluation protocol. Different text colors are used to distinguish subject, predicate, and object labels in the qualitative examples and do not indicate additional experimental settings or evaluation categories.

To further analyze the effect of SAPPM at the instance level, Figure 5 presents representative qualitative results by comparing the ground-truth annotations, the predictions of the visual-only baseline, and the predictions of SAPPM. These examples cover several spatially dependent relation types, including support relations, contact relations, relative-position relations, motion-related relations, and background-region layout relations.

Overall, the visual-only baseline tends to preserve visually salient or category-co-occurrence-based relations, such as common human–object interactions and locally evident object associations. However, when a predicate depends more strongly on spatial configurations, the corresponding subject–object pair may be missed or insufficiently ranked before relation decoding. This tendency can be observed across the representative examples in Figure 5. In the sitting scene with umbrella occlusion, the baseline captures salient hand-held relations but misses the support relation between the person and the bench. In the close-contact person–dog scene, it predicts several semantically related interactions but fails to recover the ground-truth contact relation. In the tennis scene, it captures prominent action and object-association relations, while some relations involving body posture, racket motion, and local scene layout remain incomplete. In the dog–frisbee scene, it retains object-level associations but does not fully capture motion-direction and relative-layout cues. These observations suggest that object-query feature matching alone may be insufficient for predicates that rely on mask-level spatial evidence.

In contrast, SAPPM recovers several spatially dependent relations missed by the visual-only baseline. The recovered cases include support relations under occlusion, local contact relations between interacting instances, and motion- or layout-related relations in sport scenes. These observations are consistent with the design motivation of SAPPM: mask-derived soft centroids, relative geometry, and local-neighborhood aggregation provide complementary spatial cues for candidate subject–object pair ranking, especially when the relation cannot be sufficiently determined by object categories or appearance features alone.

It should be emphasized that additional relations predicted by SAPPM but not annotated in the PSG ground truth are not treated as correct predictions in the quantitative evaluation. Since the official evaluation strictly follows the dataset annotations, these unannotated predictions are shown only as qualitative observations of visually plausible relational patterns. Therefore, they should not be interpreted as evidence beyond the ground-truth labels, but rather as illustrative cases suggesting that SAPPM may capture additional spatial configurations that are visually plausible but not counted as positive relations under the current ground-truth annotations. Overall, Figure 5 provides instance-level qualitative observations that are consistent with the quantitative results, illustrating cases in which SAPPM better retains and predicts spatially sensitive subject–object relations under the official PSG evaluation setting.

### 4.11. Internal Design of GVA and Hyperparameter Sensitivity

The performance improvement of SAPPM is closely related to the local spatial aggregation range and channel-subspace partitioning in the GVA branch. Specifically, the neighborhood size $K_{n}$ controls the range of neighboring objects involved in local-neighborhood aggregation, while the number of channel groups *G* determines the granularity of channel-subspace partitioning in Grouped Vector Attention (GVA). These two hyperparameters affect the coverage of spatial context and the manner of feature interaction, respectively, and further influence the scoring of candidate subject–object pairs before the relation decoder.

#### 4.11.1. Effect of Neighborhood Size $K_{n}$

The neighborhood size $K_{n}$ determines the number of neighboring objects aggregated by each object in GVA. A small $K_{n}$ may limit local context coverage, making it difficult for the model to sufficiently exploit support, contact, proximity, or region-reference cues related to the target object. In contrast, an excessively large $K_{n}$ may introduce weakly related objects into the attention aggregation process, thereby increasing spatial context noise. Therefore, the choice of $K_{n}$ requires a balance between effective spatial-cue coverage and suppression of irrelevant neighbors. Table 9 compares the effects of different $K_{n}$ settings on the final SGDet performance.

As shown in Table 9, enlarging the neighborhood size does not lead to monotonic performance gains. The setting $K_{n}=4$ provides the best overall trade-off across the six SGDet metrics. It achieves the highest R@20, R@100, and all mR@K scores, with R@20 and R@100 reaching 32.53 and 40.65, respectively, and mR@20, mR@50, and mR@100 reaching 27.36, 29.86, and 31.89, respectively. Although $K_{n}=8$ and $K_{n}=24$ obtain slightly higher R@50 scores, their mR@K scores are lower than those obtained with $K_{n}=4$. Since mR@K better reflects class-balanced recognition performance, this result indicates that simply expanding the local-neighborhood range does not stably improve the recognition of diverse predicate categories.

For PSG, effective relation cues are usually concentrated within a limited neighborhood that has direct spatial constraints or stable contextual associations with the target object. A larger neighborhood increases the number of object interactions, but may also introduce weakly related objects into the attention aggregation process, thereby increasing spatial context noise and weakening the discriminability of candidate subject–object pair ranking. Considering both overall recall and class-balanced recall, $K_{n}=4$ is adopted as the neighborhood size in the final SAPPM configuration.

**Figure 6:**
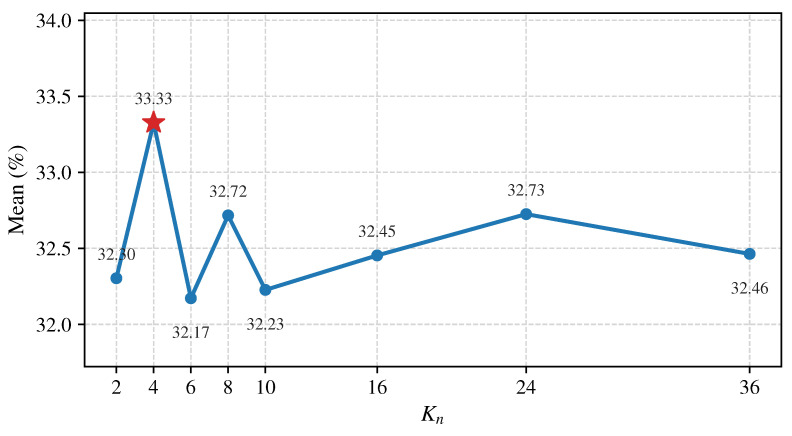
Six-metric average under different neighborhood sizes $K_{n}$. The six-metric average is computed over R@20, R@50, R@100, mR@20, mR@50, and mR@100. The red star marks the highest mean in the curve.

Figure 6 further reports the six-metric average of different $K_{n}$ settings at epoch 14. The trend is consistent with Table 9: the overall performance of GVA does not increase monotonically with $K_{n}$, and $K_{n}=4$ achieves the highest six-metric average. This indicates that the benefit of GVA mainly comes from a controlled range of local spatial interactions rather than simply expanding the neighborhood coverage.

#### 4.11.2. Effect of the Number of Channel Groups *G*

The number of channel groups *G* determines the granularity of feature-channel partitioning in GVA. In the current experimental setting, the object feature dimension is C=256; therefore, G=4, G=8, and G=16 correspond to 64, 32, and 16 channels per group, respectively. A smaller *G* preserves a larger per-group channel capacity, but different spatial–semantic interaction patterns have to share a coarser channel subspace, which may limit the ability of GVA to model differentiated neighborhood relations. Conversely, a larger *G* enables finer channel-subspace partitioning but reduces the available channel capacity of each group when the total feature dimension is fixed, which may affect the stability of joint representation between spatial cues and object semantics. Therefore, the choice of *G* requires a balance between channel-subspace diversity and per-group feature capacity.

As shown in Table 10, G=8 provides the best overall trade-off among the three settings. It achieves the highest R@20, R@50, R@100, and mR@100 scores, with values of 32.53, 37.67, 40.65, and 31.89, respectively. Compared with G=4, G=8 improves all R@K metrics and increases mR@100 from 30.93 to 31.89, indicating that a more sufficient channel-subspace partition helps the GVA model different types of spatial–semantic interactions. When *G* is further increased to 16, mR@20 and mR@50 are slightly higher than those of G=8, but R@20, R@50, R@100, and mR@100 all decrease. This result indicates that overly fine channel partitioning does not bring consistent benefits.

The above results suggest that increasing the number of channel groups is not monotonically beneficial. Instead, it introduces a trade-off between channel-subspace diversity and per-group feature capacity: a larger *G* can enhance differentiated modeling of neighborhood relations across different subspaces, but it also compresses the available feature capacity of each group and may weaken the stability of joint representation between spatial cues and object semantics. Considering both overall recall and class-balanced recall, G=8 is adopted as the number of channel groups in the final SAPPM configuration.

**Figure 7:**
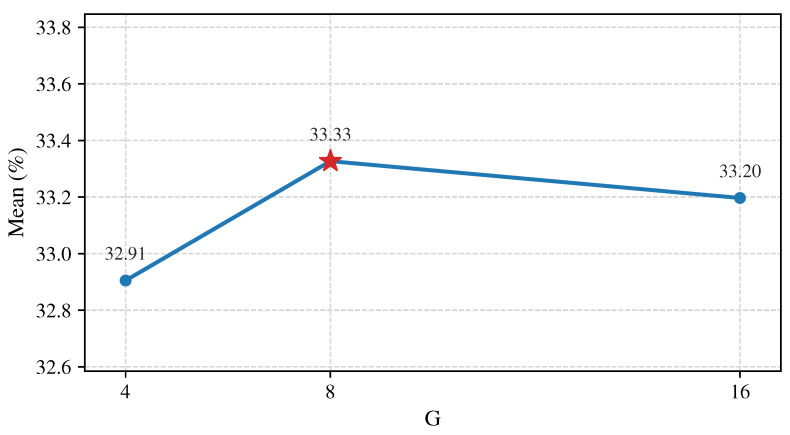
Six-metric average under different numbers of channel groups *G*. The six-metric average is computed over R@20, R@50, R@100, mR@20, mR@50, and mR@100. The red star marks the highest mean in the curve.

Figure 7 reports the six-metric average of different *G* settings at epoch 14. The result is consistent with Table 10, where G=8 achieves the highest six-metric average. This further indicates that the effectiveness of GVA does not come from simply increasing the number of channel groups, but depends on the balance between channel-subspace diversity and per-group feature capacity.

Taken together, Table 9 and Table 10 and Figure 6 and Figure 7 show that GVA exhibits non-monotonic sensitivity to both the neighborhood size $K_{n}$ and the number of channel groups *G*. Better performance does not simply result from a larger neighborhood range or finer channel partitioning but from a controlled range of local spatial interactions and an appropriate granularity of channel subspaces. This result suggests that the performance improvement of SAPPM mainly depends on effective interaction modeling among local layout, relative geometry, and object semantics, rather than indiscriminately increasing spatial context or structural complexity.

### 4.12. Mask-Quality Stratified Pair Proposal Analysis

To further analyze the proposal-stage behavior of SAPPM under different mask localization qualities, we group validation relation instances according to the mask matching quality of each ground-truth relation pair. For a ground-truth subject–object pair, the pair-level mask quality is defined as the smaller IoU of the matched subject mask and object mask:

(57) $$Q_{\mathrm{pair}}=\min\left({\mathrm{IoU}}_{s},{\mathrm{IoU}}_{o}\right).$$

This definition requires both the subject and object sides to have reliable mask matching, and therefore reflects the overall mask-level localization quality of a relation instance. We then compute proposal-stage PR@20 before relation decoding within different $Q_{\mathrm{pair}}$ ranges. The results are reported in Table 11.

As shown in Table 11, the PR@20 values of both visual path only and SAPPM increase as $Q_{\mathrm{pair}}$ becomes higher, indicating that more accurate mask localization provides a clearer spatial basis for candidate subject–object pair retention. SAPPM achieves higher PR@20 in every mask-quality range. In the 0.60–0.70, 0.70–0.80, and 0.80–0.90 ranges, SAPPM improves PR@20 by +2.53, +2.91, and +2.22 percentage points, respectively. In the highest-quality range of 0.90–1.00, SAPPM further increases PR@20 from 85.90% to 87.29%. These results show consistent proposal-stage gains across the evaluated mask-quality ranges. As masks provide clearer entity regions and spatial positions, the soft centroid, relative geometry, and local-neighborhood layout introduced by SAPPM provide effective complementary cues for candidate pair ranking, thereby improving the retention of ground-truth subject–object pairs before relation decoding.

## 5. Conclusions

This paper addresses the candidate-pair retention bottleneck in Panoptic Scene Graph Generation and proposes SAPPM, a spatially aware pair proposal model that introduces mask-level spatial structure into candidate subject–object pair scoring. Experiments on the PSG benchmark under the SGDet protocol show that SAPPM achieves competitive performance. The results indicate that incorporating mask-level spatial cues into pair proposal can improve candidate pair selection and benefit subsequent relation prediction. Nevertheless, the effectiveness of SAPPM is still partly dependent on the quality of object parsing and mask prediction, since inaccurate masks may weaken the reliability of mask-derived spatial cues.

These findings suggest that vision-sensor-based scene understanding can benefit from combining data-driven visual representation learning with explicit spatial and geometric structural modeling. Camera-captured images contain not only object appearances and semantic cues, but also mask-level object extents, relative positions, contact patterns, and local layouts. Incorporating such spatial structure into relational scene perception is relevant to sensor-based environmental perception in robotic and autonomous systems, where navigation, planning, and decision-making require an understanding of both individual objects and their spatial organization. Future work will further explore the integration of predicate semantics, language priors, and open-vocabulary relation modeling to improve PSG generalization in complex sensor-captured scenes.

## Figures and Tables

**Table 1:** SGDet main results. Mean is the arithmetic average of R@20, R@50, R@100, mR@20, mR@50, and mR@100. Published results are kept under their originally reported protocols. PSGFormer ^†^ denotes the 60-epoch PSGFormer result reported in the original PSG benchmark [8]. PSGFormer+ denotes the PSGFormer variant re-implemented with Mask2Former as the detector, as reported in Pair-Net [9]. All values are rounded to one decimal place. Bold values indicate the highest reported values in each metric column among the listed results.

Method	R@20	R@50	R@100	mR@20	mR@50	mR@100	Mean
IMP (2017) [2]	16.5	18.2	18.6	6.5	7.1	7.2	12.4
Motifs (2018) [24]	20.0	21.7	22.0	9.1	9.6	9.7	15.4
VCTree (2019) [25]	20.6	22.1	22.5	9.7	10.2	10.2	15.9
GPS-Net (2020) [27]	17.8	19.6	20.1	7.0	7.5	7.7	13.3
CIM (2024) [48]	20.7	22.1	23.8	13.4	15.0	15.3	18.4
PSGTR (2022) [8]	28.4	34.4	36.3	16.6	20.8	22.1	26.4
PSGFormer ^†^ (2022) [8]	18.0	19.6	20.1	14.8	17.0	17.6	17.9
RCFrame (2023) [13]	29.7	32.4	33.3	16.5	17.6	17.9	24.6
ADTrans (2024) [10]	26.0	29.6	30.0	26.4	29.7	30.0	28.6
PSGAtten (2025) [18]	29.1	35.2	37.3	25.1	26.2	26.6	29.9
VCTree + CAFE (2025) [12]	26.9	29.8	30.6	27.8	29.1	29.4	28.9
DGTM (2025) [17]	25.8	28.3	28.4	25.4	27.8	27.9	27.3
PSGFormer+ (2024) [9]	18.9	21.5	22.4	16.6	19.4	20.3	19.9
Pair-Net (2024) [9]	29.6	35.6	39.6	24.7	28.5	30.6	31.4
Ours	**32.5**	**37.7**	**40.7**	**27.4**	**29.9**	**31.9**	**33.4**

**Table 2:** Comparison of model scale, training overhead, inference efficiency, and estimated SAPPM spatial-path complexity. Baseline denotes the controlled Pair-Net baseline under the same codebase, frozen segmentation branch, object parsing configuration, and training protocol as SAPPM. Training Time denotes the total wall-clock training time for 14 epochs on two NVIDIA RTX 4090 GPUs with a total batch size of 8. Inference efficiency is measured for the full model under single-GPU testing with batch size 1 and is averaged over 500 validation images after 50 warm-up runs. The MACs and GFLOPs in panel (c) are estimated only for the SAPPM spatial path, where one MAC is counted as two FLOPs.

**(a) Model scale and training overhead**				
Method	Total Params	Extra Params	Training Time	Extra Training Time
Baseline	54.28 M	–	6.16 h	–
SAPPM	54.88 M	+0.60 M	6.67 h	+0.51 h
**(b) Inference efficiency under different object-query settings**				
Method	Object Queries *N*	Latency (ms)	FPS	Peak GPU Memory (GB)
Baseline	100	96.36	10.38	0.86
SAPPM	100	99.39	10.06	0.87
Baseline	200	109.02	9.17	1.03
SAPPM	200	110.81	9.02	1.03
**(c) Estimated SAPPM spatial-path complexity**				
Object Queries *N*	Spatial-Path MACs		Spatial-Path GFLOPs	
100	2.193 G		4.387	
200	8.539 G		17.079	

**Table 3:** Component ablation of SAPPM. “Visual path only” denotes the Pair-Net-style controlled baseline used in this ablation study. It uses the same frozen segmentation branch, dataset split, backbone, training schedule, batch size, augmentation strategy, and evaluation protocol as SAPPM. Bold values indicate the highest values in each metric column.

Proposal Form	R@20	R@50	R@100	mR@20	mR@50	mR@100
Visual path only	30.50	36.32	39.64	24.49	27.70	29.62
Spatial path only (GVA-enhanced)	32.08	37.30	40.23	25.92	28.44	29.98
Visual + spatial path	32.20	37.27	40.15	26.05	28.41	30.12
Visual + spatial path + gate	**32.53**	**37.67**	**40.65**	**27.36**	**29.86**	**31.89**

**Table 4:** Comparison with simplified spatial proposal variants under the SGDet setting. All variants use the same detector, relation decoder, number of selected pairs, training schedule, and SGDet evaluation protocol. Only the pair proposal module for selected pair ranking is modified. Bold values indicate the highest values in each metric column.

Method	R@20	R@50	R@100	mR@20	mR@50	mR@100
Visual path only	30.50	36.32	39.64	24.49	27.70	29.62
Visual + Geo-MLP	32.45	37.40	40.19	25.06	28.03	29.41
Visual + RPE-only	32.27	37.59	40.31	25.95	29.21	30.85
SAPPM (Visual + GVA-RPE + Gate)	**32.53**	**37.67**	**40.65**	**27.36**	**29.86**	**31.89**

**Table 5:** Effect of the spatial-anchor choice in SAPPM. All variants are evaluated under the same controlled setting. Only the spatial-anchor computation is changed, while the model structure, training schedule, query configuration, Top-$K_{r}$ candidate capacity, and SGDet evaluation protocol remain unchanged. Bold values indicate the highest values in each metric column.

Spatial Anchor	R@20	R@50	R@100	mR@20	mR@50	mR@100
Bounding-box center	32.50	37.61	40.32	24.52	27.55	29.32
Binary mask centroid	32.23	37.39	40.45	25.61	28.26	29.99
Sigmoid-based soft centroid	32.35	37.47	40.41	25.84	28.52	30.20
ReLU-logit soft centroid (ours)	**32.53**	**37.67**	**40.65**	**27.36**	**29.86**	**31.89**

**Table 6:** Relationship between pair-level recall and triplet-level recall. Pair/Proposal R@20 follows the same proposal-level pair recall protocol as PR@K. Pair/Proposal R@20 values for previous methods are taken from Pair-Net [9]. The SAPPM value is recomputed from our reproduced predictions using the same proposal-level mask-IoU matching protocol. Triplet R@20 denotes SGDet R@20. PSGFormer+ denotes the PSGFormer variant re-implemented with Mask2Former as the detector, as reported in Pair-Net [9]. Bold values indicate the highest values in each metric column.

Model	Pair/Proposal R@20	Triplet R@20
Motifs	36.7	20.0
VCTree	37.2	20.6
GPS-Net	34.3	17.8
PSGFormer	26.6	18.0
PSGFormer+	28.6	18.9
Pair-Net	52.7	29.6
SAPPM	**60.46**	**32.53**

**Table 7:** Overall pair proposal quality comparison. Values in parentheses denote gains in percentage points (pp) over visual path only.

Method	PR@20	PR@50	PR@100	mPR@20	mPR@50	mPR@100
Visual path only	57.48	69.44	75.76	67.34	76.71	80.65
SAPPM	**60.46** (+2.98 pp)	**71.15** (+1.71 pp)	**76.51** (+0.75 pp)	**69.90** (+2.56 pp)	**77.35** (+0.64 pp)	**81.16** (+0.51 pp)

**Table 8:** Distance-binned pair recall comparison. Values in parentheses denote gains in percentage points (pp) over visual path only within the same distance range. Bold values indicate the higher values within each distance group.

Method	Distance Range	PR@20	PR@50	PR@100
Visual path only	Near (d<0.3)	57.16	68.63	74.89
SAPPM	Near (d<0.3)	**60.28** (+3.12 pp)	**70.40** (+1.77 pp)	**75.70** (+0.81 pp)
Visual path only	Mid (0.3≤d<0.6)	59.13	72.73	79.13
SAPPM	Mid (0.3≤d<0.6)	**61.53** (+2.40 pp)	**74.00** (+1.27 pp)	**79.53** (+0.40 pp)
Visual path only	Far (d≥0.6)	27.59	43.10	55.17
SAPPM	Far (d≥0.6)	**37.93** (+10.34 pp)	**56.90** (+13.80 pp)	**63.79** (+8.62 pp)

**Table 9:** Effect of neighborhood size $K_{n}$ in GVA. Bold values indicate the best values in each metric column.

$K_{n}$	R@20	R@50	R@100	mR@20	mR@50	mR@100
2	31.98	37.15	40.15	25.78	28.42	30.34
4	**32.53**	37.67	**40.65**	**27.36**	**29.86**	**31.89**
6	32.28	37.52	40.45	24.96	28.26	29.56
8	32.46	**37.79**	40.48	26.10	28.93	30.54
10	32.19	37.29	40.19	25.60	28.26	29.83
16	32.14	37.27	39.83	26.06	29.04	30.38
24	32.33	37.73	40.45	26.10	29.20	30.54
36	32.03	37.22	40.17	25.72	29.16	30.48

**Table 10:** Effect of the number of channel groups *G* in GVA. Bold values indicate the best values in each metric column.

*G*	R@20	R@50	R@100	mR@20	mR@50	mR@100
4	32.17	37.58	40.36	26.73	29.66	30.93
8	**32.53**	**37.67**	**40.65**	27.36	29.86	**31.89**
16	32.33	37.50	40.43	**27.45**	**30.07**	31.40

**Table 11:** Mask-quality stratified proposal-stage Pair Recall@20. Bold values indicate the best values in each metric column.

Mask Quality $Q_{\mathrm{pair}}$	Pairs	Visual Path Only PR@20 (%)	SAPPM PR@20 (%)	Δ (%)
0.50–0.60	1033	38.53	**38.82**	+0.29
0.60–0.70	1697	51.62	**54.15**	+2.53
0.70–0.80	2646	63.57	**66.48**	+2.91
0.80–0.90	4053	75.52	**77.74**	+2.22
0.90–1.00	2085	85.90	**87.29**	+1.39

## Data Availability

SAPPM source code is available at https://github.com/hahahahavv/SAPPM (accessed on 23 June 2026). The PSG dataset is available at https://github.com/Jingkang50/OpenPSG (accessed on 23 June 2026). The trained weights and generated results are available from the corresponding author upon reasonable request.

## Appendix A. Predicate-Level Rec@100 Analysis of Representative Spatial Predicates

**Table A1:** Selection and exclusion criteria for representative spatial-relation diagnostic predicates.

Category	Predicates	Selection or Exclusion Rationale
Included: relative location/containment	in front of, beside, in	These predicates directly describe relative position or containment between the subject and object. Their labels can be judged mainly from pair-level spatial layout.
Included: contact/support/surface layout	on, on back of, standing on, sitting on, lying on, leaning on, walking on	These predicates depend on contact, support, or surface-layout relations between the subject and object. They are therefore suitable for diagnosing whether spatial pair proposal improves layout-related relations.
Included: vehicle/road-surface layout	parked on, driving on	These predicates describe vehicle–surface associations, where the relation is mainly determined by spatial support between the vehicle and the road or ground region.
Excluded: dynamic action/motion path	falling off, going down, running on, crossing, flying over, jumping over, jumping from, swinging, entering, exiting	These predicates contain spatial or directional words, but their recognition also depends strongly on action state, pose, motion path, viewpoint, depth, or temporal context. Falling off describes a transition away from a support surface, rather than a stable static spatial layout.
Excluded: appearance or fine-grained attachment	painted on, hanging from, attached to	Painted on requires local surface appearance and texture correspondence. Hanging from and attached to often depend on fine-grained physical attachment or occlusion cues that are not reliably represented by centroid-level pair geometry alone.
Excluded: broad or ambiguous spatial labels	over, enclosing	These predicates are spatially related but broad in annotation usage and can depend on viewpoint, scale, depth ordering, or object extent. They are therefore not used in the conservative representative set.
Excluded: semantic/interaction-dominant predicates	wearing, holding, and other non-spatial PSG predicates	These predicates are mainly determined by object semantics, affordance, human–object interaction, or scene co-occurrence. They are evaluated in the all-56-predicate analysis but are not representative static spatial-relation predicates.

**Table A2:** Predicate-level Rec@100 values on the representative spatial-relation diagnostic predicates. (N) denotes the number of ground-truth relationship instances for each predicate in the PSG validation set.

Predicate	(N)	Visual Path Only Rec@100	SAPPM Rec@100	Δ Rec@100
in front of	1208	31.65	34.50	+2.85
beside	1761	32.12	33.15	+1.03
in	529	27.60	31.81	+4.21
on	2631	31.44	32.04	+0.60
on back of	45	2.56	7.69	+5.13
standing on	676	52.71	52.76	+0.05
sitting on	326	39.38	41.56	+2.18
lying on	91	43.90	50.70	+6.80
leaning on	96	9.82	12.20	+2.38
walking on	249	38.15	38.96	+0.81
parked on	316	53.50	54.76	+1.26
driving on	302	66.18	66.85	+0.67
